# Alarmin IL-33 orchestrates antitumoral T cell responses to enhance sensitivity to 5-fluorouracil in colorectal cancer

**DOI:** 10.7150/thno.80483

**Published:** 2023-03-13

**Authors:** Mengjia Song, Jieying Yang, Muping Di, Ye Hong, Qiuzhong Pan, Yufei Du, Tong Xiang, Juan Liu, Yan Tang, Qijing Wang, Yongqiang Li, Jia He, Hao Chen, Jingjing Zhao, Desheng Weng, Yizhuo Zhang, Jian-Chuan Xia

**Affiliations:** 1 Collaborative Innovation Center for Cancer Medicine, State Key Laboratory of Oncology in South China, Sun Yat-sen University Cancer Center, Guangzhou, China.; 2 Department of Pediatric Oncology, Sun Yat-sen University Cancer Center, Guangzhou, China.; 3 Department of Biotherapy, Sun Yat-sen University Cancer Center, Guangzhou, China.; 4 Department of Pediatric Oncology, The Fifth Affiliated Hospital, Guangzhou Medical University, Guangzhou, China.

**Keywords:** IL-33, annexin A1, T cell response, 5-FU chemoresistance, adjuvant immunotherapy

## Abstract

**Rationale:** Resistance to 5-fluorouracil (5-FU) chemotherapy remains the main barrier to effective clinical outcomes for patients with colorectal cancer (CRC). A better understanding of the detailed mechanisms underlying 5-FU resistance is needed to increase survival. Interleukin (IL)-33 is a newly discovered alarmin-like molecule that exerts pro- and anti-tumorigenic effects in various cancers. However, the precise role of IL-33 in CRC progression, as well as in the development of 5-FU resistance, remains unclear.

**Methods:** High-quality RNA-sequencing analyses were performed on matched samples from patients with 5-FU-sensitive and 5-FU-resistant CRC. The clinical and biological significance of IL-33, including its effects on both T cells and tumor cells, as well as its relationship with 5-FU chemotherapeutic activity were examined in *ex vivo*, *in vitro* and *in vivo* models of CRC. The molecular mechanisms underlying these processes were explored.

**Results:** IL-33 expressed by tumor cells was a dominant mediator of antitumoral immunity in 5-FU-sensitive patients with CRC. By binding to its ST2 receptor, IL-33 triggered CD4+ (Th1 and Th2) and CD8+ T cell responses by activating annexin A1 downstream signaling cascades. Mechanistically, IL-33 enhanced the sensitivity of CRC cells to 5-FU only in the presence of T cells, which led to the activation of both tumor cell-intrinsic apoptotic and immune killing-related signals, thereby synergizing with 5-FU to induce apoptosis of CRC cells. Moreover, injured CRC cells released more IL-33 and the T cell chemokines CXCL10 and CXCL13, forming a positive feedback loop to further augment T cell responses.

**Conclusions:** Our results identified a previously unrecognized connection between IL-33 and enhanced sensitivity to 5-FU. IL-33 created an immune-active tumor microenvironment by orchestrating antitumoral T cell responses. Thus, IL-33 is a potential predictive biomarker for 5-FU chemosensitivity and favorable prognosis and has potential as a promising adjuvant immunotherapy to improve the clinical benefits of 5-FU-based therapies in the treatment of CRC.

## Introduction

Colorectal cancer (CRC) is the third most prevalent cancer and second leading cause of cancer-related mortality worldwide, accounting for approximately 551,000 colon cancer deaths and 310,000 rectal cancer deaths annually 1. The fluorinated analog of uracil, 5-fluorouracil (5-FU), remains the first-line chemotherapeutic option for palliative, neoadjuvant, or adjuvant treatment of CRC 2. 5-FU-based chemotherapies, such as FOLFOX (5-FU, leucovorin, and oxaliplatin) or FOLFIRI (5-FU, leucovorin, and irinotecan), have been used as the standard therapy for advanced CRC 2. However, despite advances in systemic therapies, the 5-year survival rate has not been effectively extended. Indeed, a major barrier to successful treatment is the presence of innate or acquired resistance to 5-FU in 90% of patients with CRC 3. Tumor cell-intrinsic factors such as p53 mutation, Wnt/β-catenin signaling, glucose metabolism, and oxidative stress have been implicated in the induction of 5-FU resistance 4-7. Furthermore, recent studies have reported that various immune cells in the tumor microenvironment (TME) also affect the sensitivity of 5-FU in CRC. For example, accumulation of immunosuppressive T helper (Th) 17 cells, myeloid-derived suppressor cells, and T regulatory cells, as well as a decrease in CD8^+^ T cell infiltration, have been closely associated with 5-FU resistance in patients with CRC 8-10. Therefore, a better understanding of the heterogeneity of the immune contexture and mechanisms underlying 5-FU resistance will facilitate the development of more effective targeted therapies to increase the survival benefit of 5-FU-based treatment of CRC.

Interleukin (IL)-33 is a newly discovered member of the IL-1 family of cytokines 11. When expressed in healthy cells, IL-33 functions as a nuclear factor to modulate gene expression by forming complexes with chromatin 12. Upon its release from injured cells, IL-33 transmits signals to local immune cells by binding to the suppression of tumorigenicity 2 (ST2) receptor; it is considered an alarmin-like molecule 11. Recently, multiple studies have reported the pleiotropic cytokine activities of IL-33 in mediating Th2 immune responses 13, enhancing antitumoral/antiviral Th1 and CD8^+^ T cell immunity 14-17, activating and recruiting antigen-presenting cells 18, and promoting wound healing 19 in immune disorders and various cancers. In addition, IL-33 has also been found to be closely associated with tumor progression by regulating tumor cell proliferation, apoptosis, migration, stemness, or chemoresistance in various cancers, including ovarian, colon, and breast cancers 20-22. As an endogenous danger signal, the IL-33-ST2 axis also affects other immune cells in the TME, including macrophages, mast cells, basophils, eosinophils, and specific subsets of innate lymphoid cells 23, 24. These data suggest that IL-33 may be a double-edged sword in tumor development. However, the exact role of IL-33 in CRC progression, as well as the development of 5-FU resistance, remains unclear.

Here, we performed RNA-sequencing analyses of 24 matched clinical samples from patients with 5-FU-sensitive or 5-FU-resistant CRC and examined the molecular mechanisms underlying the development of distinct sensitivities to 5-FU *ex vivo*, *in vitro* and *in vivo* models of CRC. Our data reveal a direct and functional connection between IL-33 and 5-FU sensitivity in CRC and the involvement of antitumoral T cell responses in this process. Our findings suggest that IL-33 may serve as a plausible candidate for predicting 5-FU chemosensitivity and favorable prognoses, as well as a promising adjuvant to generate effective T cell-mediated protective immunity against cancer. Specifically, the potential application of IL-33 in treating CRC may improve the efficacy of 5-FU-based therapies.

## Methods

### Ethics committee approval and patient consent

The use of human samples was approved by the Institutional Review Board of Sun Yat-Sen University Cancer Center (Approval No. B2019-023-02), and the requirement for informed consent was waived by the Institutional Review Board. All experiments involving humans were carried out in accordance with the Code of Ethics of the World Medical Association (Declaration of Helsinki). Experiments involving animals were approved by the Animal Care and Use Committee of the Sun Yat-Sen University Cancer Center (Approval No. L102012020121G).

### Patients and samples

All human samples were obtained from Sun Yat-Sen University Cancer Center. For immunohistochemical (IHC) staining, immunofluorescent staining, and enzyme-linked immunosorbent assay (ELISA), 117 paraffin-embedded tissues and 14 frozen serum samples were collected from patients with primary CRC between 2014 and 2015. For RNA sequencing, 24 fresh primary tumor tissues, adjacent peritumor tissues, and adjacent normal tissues were collected from eight patients with primary CRC after surgery. For isolating tumor-infiltrating lymphocytes (TILs) and tumor interstitial fluid (TIF), fresh CRC tissues were obtained from six patients with primary CRC. All samples in the study were confirmed by pathologic analysis. None of the patients received any therapeutic intervention such as chemotherapy and radiotherapy before surgery; however, they received 5-FU-based postoperative adjuvant chemotherapy. The inclusion criteria for patients with 5-FU-sensitive or 5-FU-resistant CRC were based on the revised The Response Evaluation Criteria In Solid Tumors guideline (version 1.1). 5-FU-sensitive patients were defined as those with regressed or stable tumor lesions that lasted for more than 4 weeks (usually evaluated by computed tomography or magnetic resonance imaging) after treatment for more than 1 month. 5-FU-resistant patients were defined as those with tumor lesions that had progressed after treatment.

### RNA-sequencing analysis

Total RNA was extracted using TRIzol and purified using the RNeasy Plus Micro Kit (Qiagen, Germany) according to the manufacturer's protocol. RNA libraries were constructed and sequenced using the OmicShare Genomic platform (Gene Denovo, China). High-quality sequence reads were aligned to a human reference genome (GRCh38). Differential expression analysis was performed using the DESeq2 R package and an absolute fold change of 2 was set as the threshold for significant differential expression. RNA-sequencing gene expression was normalized to fragments per kilobase of exon model per million mapped reads using RNA sequencing by expectation maximization.

### Isolation of fresh TILs

TILs were isolated from human fresh CRC tissues of patients who underwent surgical resection at the Sun Yat-Sen University Cancer Center. Fresh tissues were immediately stored in RPMI-1640 medium supplemented with 10% fetal bovine serum (FBS) and antibiotics for transportation on ice to the laboratory. Thereafter, the tissues were minced into small pieces and digested for 1 h at 37 °C in RPMI-1640 medium supplemented with 10% FBS and 1 mg/mL collagenase type IV (Sigma-Aldrich, Atlanta, GA, USA). After digestion, cell clumps were sieved through a 40-mm cell strainer (Falcon, Newport TN, USA) to obtain single-cell suspensions. The cells were subjected to Ficoll density gradient centrifugation, and TILs were harvested. The cells were cultured in fresh RPMI-1640 medium supplemented with 10% FBS, 100 units/mL penicillin, and 100 μg/mL streptomycin and were subsequently used for further experiments.

### Isolation of TIF from fresh tumor tissues

Fresh tissues were placed on a triple-layered 10-μm nylon mesh and spun at <50 g for 5 min to remove the surface liquid. Then, samples were centrifuged at 400 g for 10 min to isolate the TIF 25.

### Xenograft model

Female NCG mice (4 weeks of age) were purchased from the Gempharmatech Co., Ltd (Jiangsu, China). To evaluate the regulatory effects of IL-33 on T cell responses in mice and the role of annexin A1 during this process, HCT116 cells (1 × 10^7^ in 100 μL PBS) were injected subcutaneously into 20 NCG mice. After 2 weeks, the mice were randomly divided into four groups (n = 5 mice per group). Human T cells had been isolated from the peripheral blood mononuclear cells (PBMCs) of healthy donors (HD) and activated with 25 μg/mL CD3/CD28 T cell Activator (STEMCELL) and 50 U/mL rhIL-2 for 48 h. Then, T cells were transfected with the following short interfering RNAs (siRNAs): siControl, siANXA1#1, and siANXA1#2. Mice were intravenously injected with human T cells (1 × 10^7^ in 200 μL PBS). The mice injected with siControl-transfected T cells were treated with PBS or recombinant human (rh) IL-33 (100 μg/kg intraperitoneal injection once every other day; Peprotech, USA) between weeks 2 and 3. The mice injected with siANXA1#1-transfected and siANXA1#2-transfected T cells were treated with rhIL-33 (10 mg/kg intraperitoneal injection once every other day; Peprotech) between weeks 2 and 3. The mice were sacrificed at week 3, and their tumors were harvested. Flow cytometry was used to assess IL-2, IFN-γ, T-bet, IL-4, IL-10, and GATA3 levels in CD4^+^ T cells, and IFN-γ, TNF-α, and Ki-67 levels in CD8^+^ T cells.

To examine the effects of IL-33-mediated T cell responses on the antitumor activity of 5-FU in mice, luciferase-infected HCT116 cells (1 × 10^7^ cells in 100 μL PBS) were injected subcutaneously into 30 NCG mice. After 10 days, the mice were randomly divided into six groups (n = 5 per group). Human T cells had been isolated from the PBMCs of healthy donors (HD) and activated with 25 μg/mL CD3/CD28 T cell Activator (STEMCELL) and 50 U/mL rhIL-2 for 48 h. Then, mice were intravenously injected with or without human T cells (1 × 10^7^ in 200 μL PBS) at days 0 and 10. rhIL-33 (100 μg/kg intraperitoneal injection once every other day; Peprotech) were or were not administrated from days 0 to 20. Three days after the first T cell infusion, mice were or were not treated with 5-FU (10 mg/kg/day intraperitoneal injection; Sigma-Aldrich) until days 20. Tumor growth was monitored every week using an *in vivo* imaging system (PerkinElmer, IVIS Lumina Series III, USA). Mice were sacrificed when the diameter of the xenograft tumor reached 20 mm in any dimension or 60 days after the first T cell infusion. Xenografts were harvested and subjected to quantitative polymerase chain reaction (qPCR) analysis to determine *IL-33*, *CXCL10*, and *CXCL13* expression levels and IHC staining to assess CD3, CD206, and cleaved-caspase 3 expression.

### Murine CT26 colon cancer model

To evaluate the effect of IL-33 on 5-FU chemotherapy, 20 female BALB/c mice (4 weeks of age) were purchased from Gempharmatech Co., Ltd (Jiangsu, China). CT26 cells (3 × 10^5^ cells in 100 μL PBS) were injected subcutaneously into 20 BALB/c mice. After 7 days, the tumor size reached about 50 mm^3^, and the mice were randomly divided into four groups (n = 5 per group). The mice were treated with recombinant murine IL-33 (100 μg/kg intraperitoneal injection once every other day; Peprotech) from days 0 to 17. After 3 days, the mice were or were not treated with 5-FU (10 mg/kg/day intraperitoneal injection; Sigma-Aldrich) from days 3 to 17. Tumor size was measured every 4 days. Mice were sacrificed on day 21, and the tumor volume was calculated using the following formula: (length × width^2^)/2. Mice were sacrificed when the diameter of the tumor reached 20 mm in any dimension or 60 days after the first recombinant murine IL-33 treatment. Tumors were harvested and subjected to qPCR analysis to determine *IL-33*, *CXCL10*, and *CXCL13* expression levels and IHC staining to assess CD3, CD206, and cleaved-caspase 3 expression.

## Results

### High IL-33 expression is closely correlated with 5-FU sensitivity and type 1 and type 2 immune responses in patients with CRC

**Figure 1:**
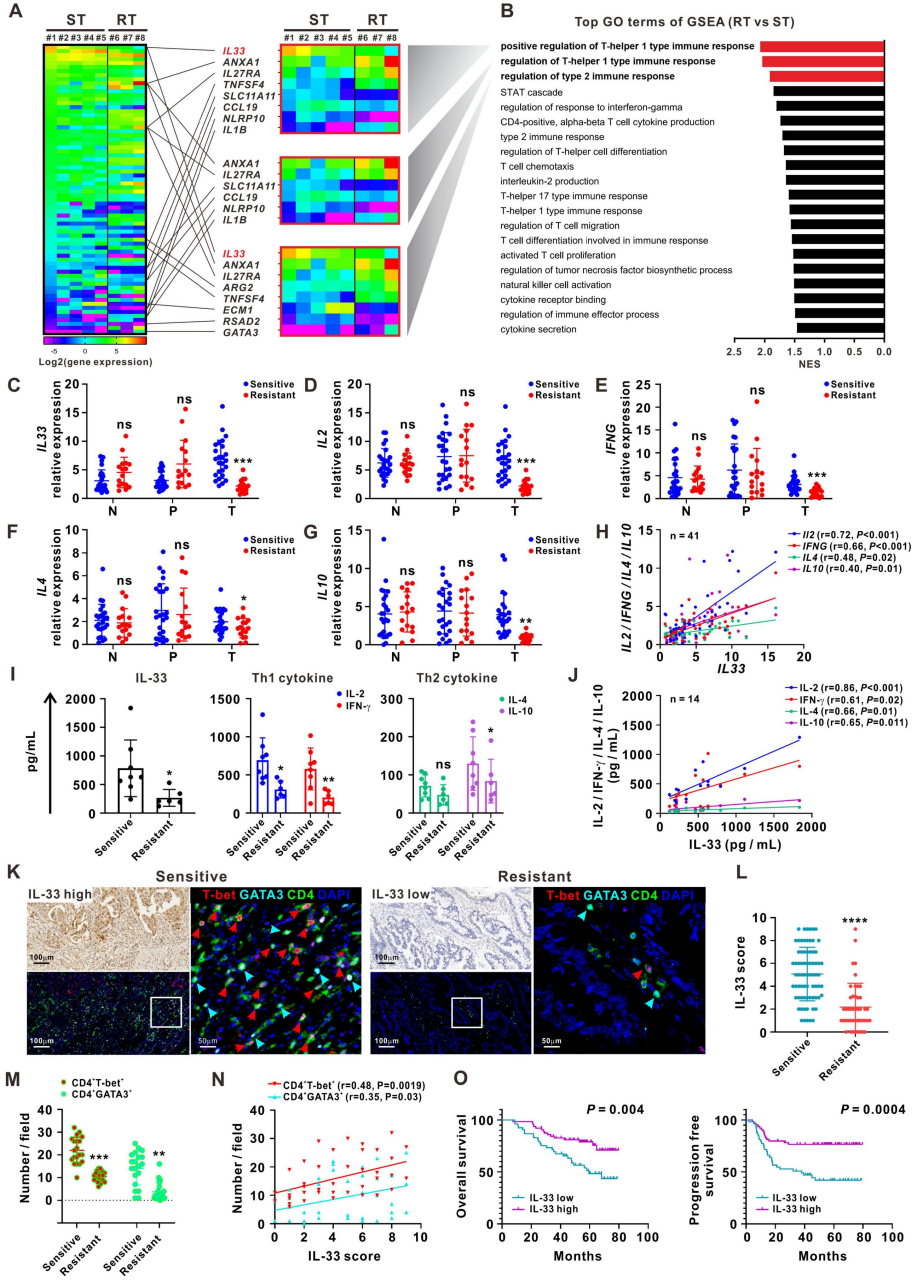
** High IL-33 expression is closely correlated with 5-FU sensitivity, and type 1 and type 2 immune responses in CRC patients.** (A) Heatmap depicting FPKM values for DEGs between 5-FU-sensitive (n = 5) and -resistant (n = 3) tumor tissues from CRC patients by RNA-sequencing. ST: sensitive tumor, RT: resistant tumor. (B) Bar plot ranking of the top GO terms according to GSEA of DEGs. (C-G) qPCR analysis for the mRNA expression *IL33* and Th1 (*IL2* and *IFNG*) and Th2 (*IL4* and *IL10*) cytokines in 123 matched samples from 41 CRC patients, including normal, peritumor, and tumor tissues from 5-FU-sensitive (n = 25) and resistant (n = 16) patients. N: normal tissues; P: peritumor tissues; T: tumor tissues. (H) Correlation between the mRNA expression of *IL33* and Th1 (*IL2* and *IFNG*) and Th2 (*IL4* and *IL10*) cytokines in 41 CRC tissues was analyzed by Pearson's correlation method. (I) The expression levels of IL-33 and Th1- and Th2-related cytokines in the serum of 5-FU-sensitive (n = 8) and resistant (n = 6) CRC patients were detected by ELISA. (J) Correlation of IL-33 with Th1 and Th2-related cytokines in the serum of 14 CRC patients was analyzed by Pearson's correlation method. (K) Consecutive sections of 5-FU-sensitive and -resistant tumor tissues from CRC patients were used to analyze the expression IL-33 by IHC staining and the co-expression pattern of T-bet (red), GATA3 (cyan), CD4 (green), and DAPI (blue) by multiplex immunofluorescence staining. Figure panel pairs the representative images taken with different zooming options. Scale bar, 100 μm or 50 μm. (L) The scores of IL-33 based on IHC staining in 5-FU-sensitive (n = 72) and -resistant (n = 45) tumor tissues are shown as a statistical graph. (M) The number of CD4^+^ T-bet^+^ and CD4^+^GATA3^+^ cells per field based on multiplex immunofluorescence staining in 5-FU-sensitive (n = 20) and -resistant (n = 20) tumor tissues are shown as a statistical graph. (N) Correlation of IL-33 score with the number of CD4^+^ T-bet^+^ and CD4^+^GATA3^+^ cells were analyzed by Pearson's correlation method in 40 CRC tissues. (O) Kaplan-Meier curves for overall survival and progression free survival in 117 CRC patients with low and high IL-33 expression based on the results of IHC staining. Data are representative of three independent experiments. *P < 0.05, **P < 0.01, ***P < 0.001, ****P < 0.0001.

RNA-sequencing analyses were first performed on 24 matched clinical samples from patients with 5-FU-sensitive or 5-FU-resistant CRC. The samples included primary tumor, peritumor, and normal tissues. A greater number of differentially expressed genes (DEGs) was observed in tumor tissues than in peritumor and normal tissues (**Figure S1A-B**). The DEGs between 5-FU-sensitive and 5-FU-resistant tumor tissues were mainly enriched in the Gene Ontology (GO) term “regulation of Th1 and Th2 type immune responses” by gene-set enrichment analysis (GSEA). IL-33 was the most significant DEG involved in these processes and was significantly upregulated in 5-FU-sensitive tumor tissues (**Figure 1A-B, Figure S1C**). Both RNA sequencing and qPCR analysis of samples from an enlarged cohort consisting of 41 patients with CRC showed that the expression of *IL33* and its receptor *ST2* was higher in tumor tissues than in peritumor and normal tissues, and a higher trend of *IL33* expression by tumor tissues was also observed in late-stage patients (**Figure S1D-G**). Immune profiling analysis based on the RNA-sequencing data showed a higher expression level of “T cells CD4 memory activated” and “T cells CD8” (although not statistically significant) in 5-FU-sensitive tissues than in 5-FU-resistant tissues, further supporting our research focus on T cell response in 5-FU chemosensitivity (**Figure S1H**).

IL-33 is a novel member of the IL-1 family of cytokines and has been implicated in the modulation of inflammatory disorders such as asthma, atopic dermatitis, and some cancers 11-17. However, the precise role of IL-33 in CRC remains unclear. We further collected 123 matched samples from 41 patients with CRC for validation. The qPCR analyses showed higher expression levels of *IL33*, Th1 cytokines (*IL2* and *IFNG),* and Th2 cytokines (*IL4* and *IL10*) in 5-FU-sensitive tumor tissues than in 5-FU-resistant tumor tissues, whereas the expression levels of these cytokines did not significantly differ between normal and peritumor tissues (**Figure 1C-G**). Furthermore, *IL33* expression levels were positively correlated with those of Th1 cytokines (*IL2* and *IFNG)* and Th2 cytokines (*IL4* and *IL10*) in 41 tumor tissues (**Figure 1H**). Similar results were observed using the sera of patients with CRC (**Figure 1I-J**).

Next, we used IHC staining to examine the distribution pattern of IL-33 and T cell infiltration in different regions of tissues obtained from patients with CRC (**Figure S2A-B**). Interestingly, higher levels of IL-33 expression and CD3^+^ T cell infiltration were found in tumoral but not peritumoral or edge regions of tissues from 5-FU-sensitive patients than from 5-FU-resistant patients, consistent with our RNA-sequencing data. Furthermore, higher densities of tumor-infiltrating CD3^+^ T cells were observed migrating from the peritumoral and edge regions of tumors from 5-FU-sensitive patients than from 5-FU-resistant patients (**Figure S2A-B**). This phenomenon was consistent with the GSEA data based on the RNA-sequencing data showing enrichment of the GO terms “T cell chemotaxis” and “regulation of T cell migration” in 5-FU-sensitive tumors (**Figure 1B**), as well as the qPCR data showing significant upregulation of the T cell chemokines *CXCL10* and *CXCL13* in 5-FU-sensitive tumors (**Figure S2C**). Accordingly, we also found a positive correlation between IL-33 expression and CD3^+^ T cell infiltration in CRC tumor tissues (**Figure S2D**). In addition, multiplex immunofluorescence staining of consecutive tissue sections revealed that CD4^+^ T-bet^+^ (Th1) and CD4^+^GATA3^+^ (Th2) cells displayed a simultaneously high and low expression pattern of IL-33 in 5-FU-sensitive and 5-FU-resistant patients, respectively (**Figure 1K-M**). The level of IL-33 was positively associated with the densities of both CD4^+^ T-bet^+^ (Th1) and CD4^+^GATA3^+^ (Th2) cells (**Figure 1N**), suggesting a potential role of IL-33 in modulating Th1 and Th2 T cell responses in 5-FU-sensitive patients.

Survival analysis based on the IHC staining data revealed that patients with high levels of IL-33 expression had longer overall survival and progression-free survival (**Figure 1O**). Multivariate Cox regression analysis indicated that IL-33 was an independent prognostic indicator for the overall survival and progression-free survival of patients with CRC (**Table 1**). Together, these data suggest that IL-33 is positively associated with 5-FU sensitivity and may play a potential role in modulating T cell responses in patients with CRC.

### IL-33 secreted by CRC cells drives Th1 and Th2 T cell responses through interaction with its receptor ST2

**Figure 2:**
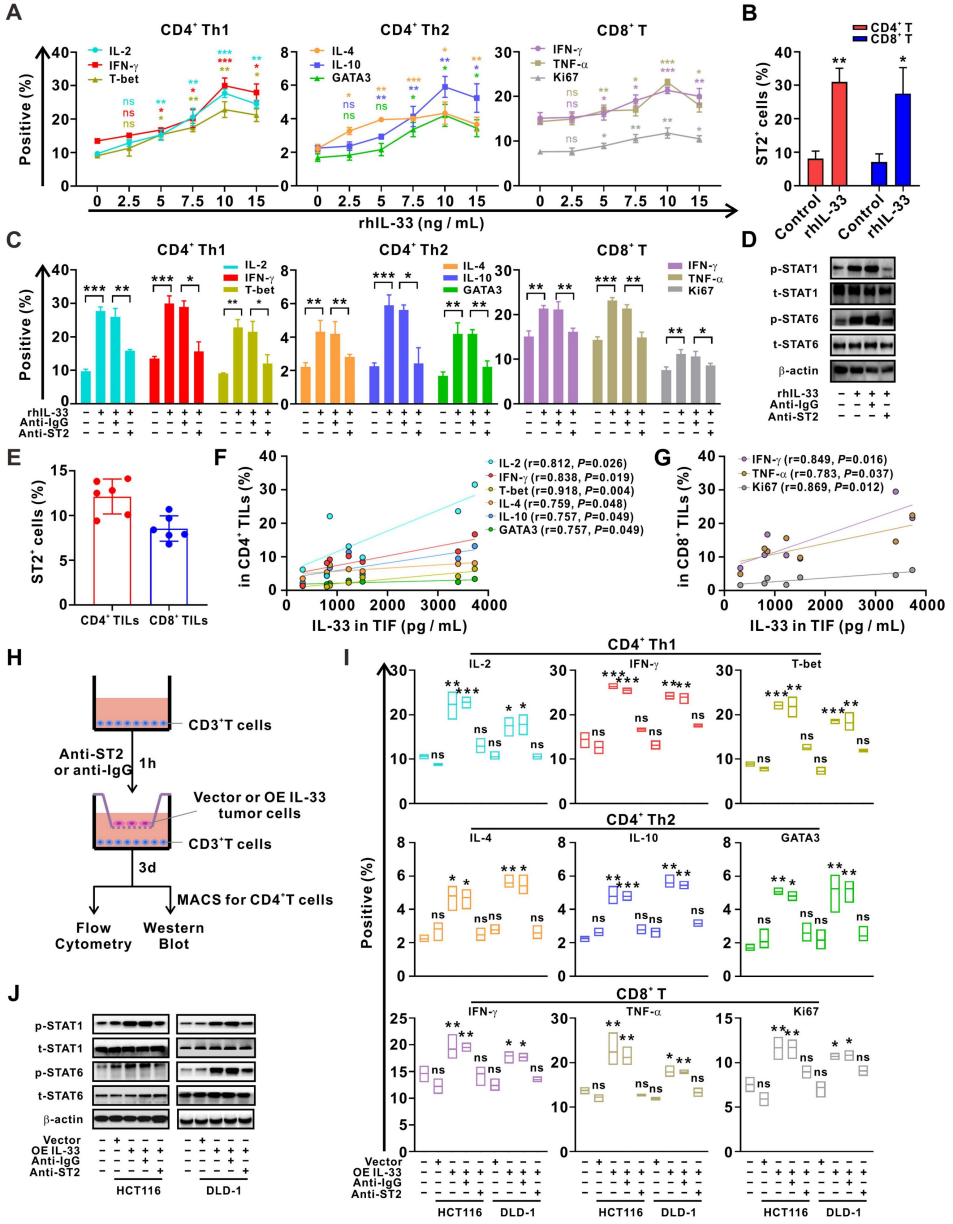
**IL-33 secreted by CRC cells drives T cell Th1 and Th2 responses through interaction with its receptor ST2**. (A) CD3^+^ T cells isolated from the PMBCs of HD were activated with CD3/CD28 T cell Activator for 48 h and then treated with different doses of recombinant human IL-33 for 72 h. The intracellular expression of IL-2, IFN-γ, IL-4, and IL-10 and nuclear expression of T-bet and GATA3 by CD4^+^ T cells and intracellular expression of IFN-γ and TNF-α and nuclear expression of Ki67 by CD8^+^ T cells were examined by flow cytometry (n = 3). (B) CD3^+^ T cells from the PBMCs were treated with or without rhIL-33 (10 ng/mL) for 72 h. The surface expression of ST2 by CD4^+^ and CD8^+^ T cells were examined by flow cytometry (n = 3). (C-D) CD3^+^ T cells from the PBMCs were pretreated with anti-IgG or anti-ST2 neutralizing antibodies (1 μg/mL) for 1 h, followed by rhIL-33 (10 ng/mL) treatment for 72 h. The intracellular expression of IL-2, IFN-γ, IL-4, and IL-10 and nuclear expression of T-bet and GATA3 by CD4^+^ T cells and intracellular expression of IFN-γ and TNF-α and nuclear expression of Ki67 by CD8^+^ T cells were examined by flow cytometry (n = 3) (C). CD4^+^ T were further isolated using MACS magnetic sorting system, and western blot analysis was performed for the expression of phospho-STAT1, total STAT1, phospho-STAT6, total STAT6, and β-actin in CD4^+^ T cells (D). (E-G) TILs and TIF were isolated from fresh tumor tissues of 6 CRC patients. The surface expression of ST2 by CD4^+^ TILs and CD8^+^ TILs was examined by flow cytometry (E). The concentration of IL-33 in TIF was detected by ELISA. The intracellular expression of IL-2, IFN-γ, IL-4, and IL-10, and nuclear expression of T-bet and GATA3 in CD4^+^ TILs were examined by flow cytometry; the surface expression of annexin A1, intracellular expression of IFN-γ and TNF-α, and nuclear expression of Ki67 in CD8^+^ TILs were examined by flow cytometry; correlation analyses were performed using Pearson's correlation method (F, G). (H-J) CD3^+^ T cells from the PBMCs of HD were pretreated with anti-IgG or anti-ST2 neutralizing antibodies (1 μg/mL) for 1 h, respectively, following by coculture with CRC cells with or without IL-33 overexpression for 72 h in Transwell system (H). The intracellular expression of IL-2, IFN-γ, T-bet, IL-4, and IL-10 and nuclear expression of GATA3 by CD4^+^ T cells and IFN-γ, TNF-α, and Ki67 by CD8^+^ T cells were examined by flow cytometry (n = 3) (I). CD4^+^ T were further isolated using MACS magnetic sorting system, and western blotting analysis for the expression of phospho-STAT1, total STAT1, phospho-STAT6, total STAT6, and β-actin in CD4^+^ T cells (J). rhIL-33, recombinant human IL-33; ns, no significance. Data are representative of three independent experiments. *P < 0.05, **P < 0.01, ***P < 0.001.

To clarify the effects of IL-33 on T cell response, CD3^+^ T cells were isolated from PBMCs of HD and were treated with different doses of rhIL-33 *in vitro*. We found that IL-33 augmented the Th1 and Th2 responses of CD4^+^ T cells by inducing their differentiation in a dose-dependent manner, peaking at 10 ng/mL (**Figure 2A**). IL-33 also enhanced the CD8^+^ T cell response by increasing their effector function and proliferation (**Figure 2A**). Similar effects on ST2 expression levels were observed (**Figure S3**), which were further confirmed by flow cytometry (**Figure 2B**). However, the effect of IL-33 on enhancing T cell responses was significantly blocked by ST2-neutralizing antibodies (**Figure 2C**). In addition, similar results were obtained upon activation of STAT1 and STAT6 in CD4+ T cells (**Figure 2D**), which have been extensively implicated in the Th1 and Th2 differentiation of CD4+ T cells, respectively 26.

TILs and TIF were isolated from the fresh tumor tissues of patients with CRC. It was found that approximately 15% CD4^+^ TILs and 10% CD8^+^ TILs were expressing ST2 (**Figure 2E**). Moreover, the concentration of IL-33 in TIF was positively correlated with the frequencies of IFN-γ^+^CD4^+^, IL-2^+^CD4^+^, T-bet^+^CD4^+^, IL-4^+^CD4^+^, IL-10^+^CD4^+^, and GATA3^+^CD4^+^ TILs (**Figure 2F**), as well as those of IFN-γ^+^CD4^+^, TNF-α^+^CD8^+^, and Ki67^+^CD8^+^ TILs (**Figure 2G**), which supports the role of IL-33 in regulating Th1 and Th2 responses. Multiplex immunofluorescence staining revealed that ST2 was co-expressed with T cells and tumor cells (**Figure S4**), and that IL-33 was predominantly derived from tumor cells in the TME of CRC tissue (**Figure S5A**). To verify the role of tumor cell-derived IL-33 in T cell responses, we constructed IL-33-overexpressing CRC cell lines (**Figure S5B-C**) and co-cultured these cells with T cells from PBMCs (**Figure 2H**). The results demonstrated that both the Th1 and Th2 responses of CD4^+^ T cells, as well as the CD8^+^ T cell response, were enhanced after IL-33 was overexpressed in CRC cells. In contrast, this phenomenon was reversed by blocking ST2 in the co-culture system (**Figure 2I-J**). These findings suggest that IL-33 secreted by CRC cells interacts with its receptor ST2 and amplifies both CD4^+^ (Th1 and Th2) and CD8^+^ T cell responses.

### Annexin A1 is crucial for IL-33-mediated enhancement of T cell responses

**Figure 3:**
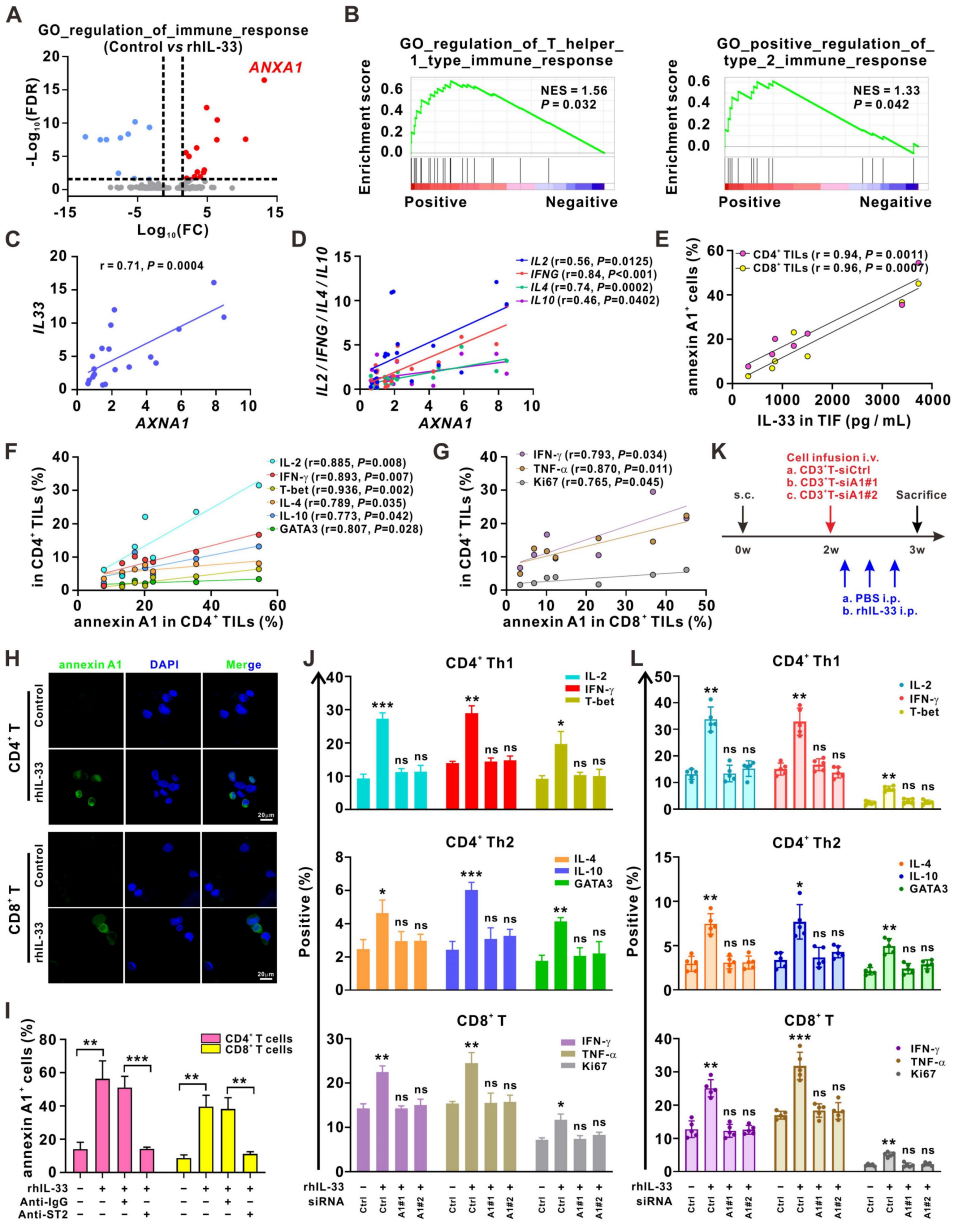
** Annexin A1 mediates T cell responses induced by IL-33.** (A) 2×10^6^ CD3^+^ T cells isolated from the PMBCs of HD were activated with CD3/CD28 T cell Activator for 48 h and then treated with rhIL-33 (10 ng/mL) for 72 h. RNA-sequencing was performed and the differentially expressed genes in the GO term of “regulation of immune response” were shown as volcano plots. (B) GSEA plot showed that annexin A1 expression was positively correlated with the regulation of Th1- and Th2- type immune response in The Cancer Genome Atlas database of CRC. (C, D) Correlation between the mRNA expression of *ANXA1* and *IL33* (C) and Th1 and Th2-related cytokines (D) in 20 tumor tissues from CRC patients was evaluated by Pearson's correlation method. (E-G) TILs and TIF were isolated from fresh tumor tissues of 6 CRC patients. The concentration of IL-33 in TIF was detected by ELISA. The surface expression of annexin A1, intracellular expression of IL-2, IFN-γ, IL-4, and IL-10, and nuclear expression of T-bet and GATA3 in CD4^+^ TILs of CRC patients were examined by flow cytometry; the surface expression of annexin A1, intracellular expression of IFN-γ and TNF-α, and nuclear expression of Ki67 in CD8^+^ TILs of CRC patients were examined by flow cytometry; correlation analyses were performed using Pearson's correlation method. (H) CD4^+^ T and CD8^+^ T isolated from the PMBCs of HD were activated with CD3/CD28 T cell Activator for 48 h and then treated with rhIL-33 (10 ng/mL) for 72 h. Cell immunofluorescent staining of annexin A1. Scale bar, 20 μm. (I) CD3^+^ T cells from the PMBCs of HD were activated with CD3/CD28 T cell Activator for 48 h. Then, CD3^+^ T cells were pretreated with anti-IgG or anti-ST2 neutralizing antibodies (1 μg/mL) for 1 h, followed by rhIL-33 (10 ng/mL) treatment for 72 h. The surface expression of annexin A1 by CD4^+^ T cells and CD8^+^ T cells was examined by flow cytometry. (J) CD3^+^ T cells from the PMBCs of HD were activated with CD3/CD28 T cell Activator for 48 h and transfected with siControl, siANXA1#1, or siANXA1#2 RNA following rhIL-33 (10 ng/mL) treatment for 72 h. Flow cytometry analysis for the intracellular expression of indicated proteins. (K) Experimental scheme for the subcutaneous xenograft model of CRC in NCG mice. Mice are inoculated with 1×10^7^ HCT116 cells subcutaneously. 1×10^7^ CD3^+^ T cells were isolated from PBMCs of HD and infused to mice after 2 weeks. rhIL-33 (10 mg/kg intraperitoneal injection, once every other day) were administrated between weeks 2 and 3. (L) Flow cytometry analysis for the intracellular expression of indicated proteins by CD4^+^ T and CD8^+^ T cells in mice-bearing xenografts. HD, healthy donors; rhIL-33, recombinant human IL-33; TIF: tumor interstitial fluid; ns, no significance. Data are representative of three independent experiments. *P < 0.05, **P < 0.01, ***P < 0.001.

Next, we examined the molecular mechanism underlying IL-33-mediated induction of T cell responses. The RNA-sequencing analysis demonstrated that *ANXA1*, a gene encoding the membrane-localized protein of annexin A1, was the most significantly upregulated gene in the GO term “regulation of immune response” after the CD3^+^ T cells were stimulated with rhIL-33 (**Figure 3A**). Annexin A1 is a calcium- and phospholipid-binding protein expressed by many cell types that plays a role in the inflammatory response, as well as in innate and adaptive immunity 27. As shown in **Figure 1A-B**, *ANXA1* was involved in “regulation of Th1 and Th2 type immune responses,” which was enriched in 5-FU-sensitive tumors. The GSEA analysis of the CRC data from The Cancer Genome Atlas dataset also suggested a strong association between *AXNA1* and the GO term “regulation of Th1 and Th2 type immune responses” (**Figure 3B**). Consistently, we observed a positive correlation between *AXNA1* and both *IL-33* (**Figure 3C**) and Th1/Th2 cytokines (**Figure 3D**) in CRC tissues. Moreover, the concentration of IL-33 in TIF showed a significantly positive association with the frequencies of annexin A1^+^CD4^+^ TILs and annexin A1^+^CD8^+^ TILs (**Figure 3E**); the frequencies of annexin A1^+^CD4^+^ TILs were positively correlated with those of IFN-γ^+^CD4^+^, IL-2^+^CD4^+^, T-bet^+^CD4^+^, IL-4^+^CD4^+^, IL-10^+^CD4^+^, and GATA3^+^CD4^+^ TILs (**Figure 3F**). A positive correlation between the frequencies of annexin A1^+^CD8^+^ TILs and IFN-γ^+^CD4^+^, TNF-α^+^CD8^+^, and Ki67^+^CD8^+^ TILs was also observed (**Figure 3G**). These data suggest the potential of annexin A1 to mediate IL-33-induced T cell responses in CRC. Indeed, immunofluorescence colocalization and flow cytometry analyses demonstrated that annexin A1 expression was significantly upregulated by IL-33 in both CD4^+^ and CD8^+^ T cells from PBMCs (**Figure 3H-I**). Based on these findings, we hypothesized that annexin A1 might be required for the IL-33-mediated induction of the Th1 and Th2 responses of T cells.

To test this hypothesis, we silenced annexin A1 expression in T cells from PBMCs of HD using siRNAs. The results showed that annexin A1 knockdown effectively reversed the CD4^+^ (Th1 and Th2) and CD8^+^ T cell responses induced by IL-33 *in vitro* (**Figure 3J**). A xenograft model of CRC was established in NCG mice for further validation (**Figure 3K**). CD3^+^ T cells isolated from PBMCs of human HD were or were not transfected with ANXA1 siRNAs. Subsequently, these CD3^+^ T cells were infused into mice, and rhIL-33 was administrated. Flow cytometry analyses of the infused T cells in the mouse peripheral blood revealed that rhIL-33 administration enhanced the CD4^+^ (Th1 and Th2) and CD8^+^ T cell responses in mice infused with control CD3^+^ T cells; however, this phenomenon was not observed in mice infused with siANXA1 CD3^+^ T cells (**Figure 3L**). Taken together, our results indicate that IL-33 mediates T cell responses by upregulating annexin A1.

### Activation of JAK-STAT and p38-p65 signaling by annexin A1 is partially required for IL-33-induced Th1 and Th2 responses in CD4^+^ T cells

**Figure 4:**
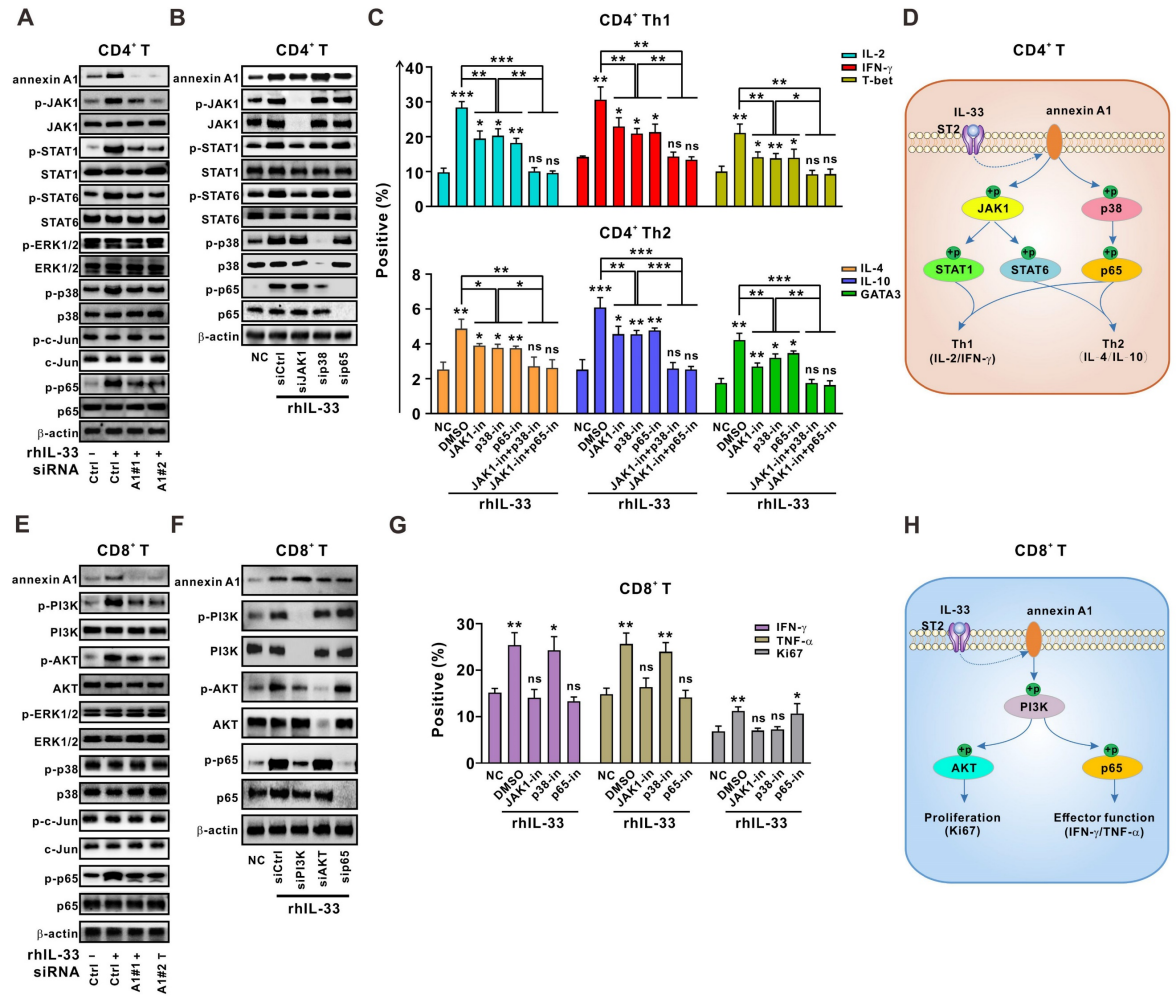
** Annexin A1 downstream signaling cascades are partially responsible for IL-33-induced Th1 and Th2 responses.** (A) CD4^+^ T cells from the PMBCs of HD were activated with CD3/CD28 T cell Activator for 48 h and transfected with siControl, siANXA1#1, or siANXA1#2 RNA following rhIL-33 (10 ng/mL) treatment for 72 h. Western blot analysis was performed for the expression of indicated proteins. (B, C) CD4^+^ T cells from the PMBCs of HD were activated with CD3/CD28 T cell Activator for 48 h and transfected with siControl, siJAK1, sip38, or sip65 RNA following rhIL-33 (10 ng/mL) treatment for 72 h. Western blot analysis was performed for the expression of indicated proteins (B). The intracellular expression of IL-2, IFN-γ, T-bet, IL-4, IL-10, and GATA3 by CD4^+^ T cells were examined by flow cytometry (C). (D) Proposed model showing annexin A1-involved signaling pathways in CD4^+^ T educated by IL-33. (E) CD8^+^ T cells from the PMBCs of HD were activated with CD3/CD28 T cell Activator for 48 h and transfected with siControl, siANXA1#1, or siANXA1#2 RNA following rhIL-33 (10 ng/mL) treatment for 72 h. Western blot analysis was performed for the expression of indicated proteins. (F, G) CD8^+^ T cells from the PMBCs of HD were activated with CD3/CD28 T cell Activator for 48 h and transfected with siControl, siJAK1, sip38, or sip65 RNA following rhIL-33 (10 ng/mL) treatment for 72 h. Western blot analysis was performed for the expression of indicated proteins (F). The intracellular expression of IFN-γ, TNF-α, and Ki67 by CD8^+^ T cells were examined by flow cytometry (G). (H) Proposed model showing annexin A1-involved signaling pathways in CD8^+^ T educated by IL-33. HD, healthy donors; rhIL-33, recombinant human IL-33; ns, no significance. Data are representative of three independent experiments. *P < 0.05, **P < 0.01, ***P < 0.001.

We next sought to determine how IL-33-induced annexin A1 regulates the T cell response. Previous studies have shown that JAK1-STAT1 and JAK1-STAT6 signaling pathways are involved in Th1 and Th2 differentiation of CD4^+^ T cells, respectively 26. Mitogen-activated protein kinase (MAPK) signaling, including ERK1/2, p38, JNK, and NF-κB signaling, has been shown to play a role in CD4^+^ T cell activation and differentiation in antitumoral or antiviral immunity 26. We found that JAK1-STAT1/STAT6, p38, and p65 signaling pathways were activated by IL-33 in CD4^+^ T cells from the PBMCs of HD, whereas annexin A1 silencing partially abolished this response (**Figure 4A**). However, JAK1, p38, and p65 silencing did not affect annexin A1 expression (**Figure 4B**). Therefore, our results suggest that JAK1-STAT1/STAT6, p38, and p65 pathways are downstream of annexin A1 in CD4^+^ T cells.

As shown in **Figure 4B**, JAK1 silencing in CD4^+^ T cells led to a significant reduction in STAT1 and STAT6 activation but did not affect p38 and p65 activation, suggesting that STAT1 and STAT6 are downstream of JAK1. Silencing of p38 resulted in an obvious reduction in p65 activation, whereas silencing of p65 did not affect the activation of other pathways, suggesting that p65 is downstream of p38 in CD4^+^ T cells. Functional experiments (**Figure 4C**) demonstrated that the knockdown of JAK1 synergized with p38 and p65 silencing to abrogate the IL-33-induced Th1 and Th2 responses of CD4^+^ T cells. Taken together, our data indicate that the JAK1-STAT1/STAT6 and p38-p65 pathways are downstream signaling targets of annexin A1 and partially mediate the IL-33-induced Th1 and Th2 responses of CD4^+^ T cells (**Figure 4D**).

### Activation of PI3K-AKT/p65 signaling pathways by annexin A1 is partially responsible for the IL-33-mediated CD8^+^ T cell response

Similar methods were used to determine the role of annexin A1 in the IL-33-induced CD8^+^ T cell response. As shown in **Figure 4E**, the knockdown of annexin A1 in CD8^+^ T cells from the PBMCs of HD partially mitigated the phosphorylation of PI3K-AKT and p65 signaling pathways activated by IL-33, whereas silencing of PI3K, AKT, and p65 did not affect annexin A1 expression, suggesting that PI3K-AKT and p65 pathways are downstream of annexin A1 in CD8^+^ T cells.

PI3K knockdown in CD8^+^ T cells led to reduced activation of AKT and p65. However, neither AKT nor p65 silencing affected the activation of other pathways (**Figure 4F**). Thus, AKT and p65 are downstream of PI3K in CD8^+^ T cells. Interestingly, functional data on CD8^+^ T cells (**Figure 4G**) demonstrated that PI3K knockdown significantly reversed the IL-33-mediated increase in cytokine production and Ki-67 expression, whereas p65 silencing only reversed cytokine production and AKT silencing only reversed Ki-67 expression. These results suggest that PI3K-AKT and PI3K-p65 signaling pathways activated by annexin A1 are partially responsible for the IL-33-mediated effector function and proliferation of CD8^+^ T cells, respectively (**Figure 4H**).

### IL-33 enhances the sensitivity of CRC cells to 5-FU in a T cell-dependent manner

**Figure 5:**
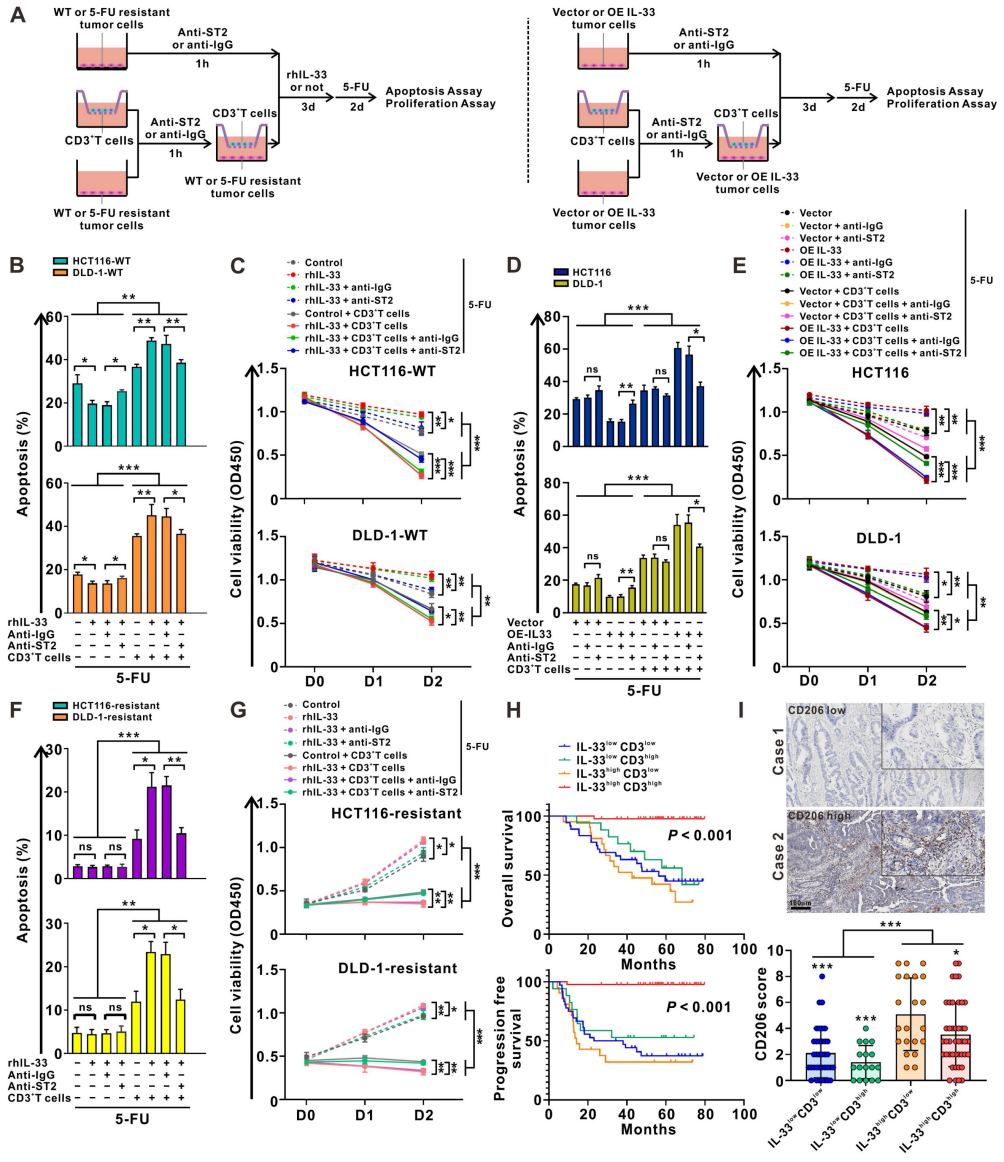
**IL-33 enhances the sensitivity of CRC cells to 5-FU in a T cell-dependent manner.** (A) Graphical representation of the various coculture conditions of CRC cells and CD3^+^ T cells isolated from the PMBCs of HD. (B, C) Wild type CRC cells and CD3^+^ T cells were pretreated with anti-IgG or anti-ST2 neutralizing antibodies (1 μg/mL), respectively. Then, CRC cells were alone cultured or cocultured with CD3^+^ T cells with or without rhIL-33 (10 ng/mL) treatment, followed by 5-FU (10 μg/mL) treatment. After 48 h, cell apoptosis was measured by flow cytometry analysis (B). Cell viability was evaluated at indicated time points by CCK-8 assay (C). (D-E) CRC cells with or without IL-33 overexpression and CD3^+^ T cells were pretreated with anti-IgG or anti-ST2 neutralizing antibodies (1 μg/mL), respectively. Then, CRC cells were alone cultured or cocultured with CD3^+^ T cells, followed by 5-FU (10 μg/mL) treatment. After 48 h, cell apoptosis was measured by flow cytometry analysis (D). Cell viability was evaluated at indicated time points by CCK-8 assay (E). (F-G) 5-FU-resistant CRC cells and CD3^+^ T cells were pretreated with anti-IgG or anti-ST2 neutralizing antibodies (1 μg/mL), respectively. Then, CRC cells were alone cultured or cocultured with CD3^+^ T cells with or without recombinant human IL-33 (10 ng/mL) treatment, followed by 5-FU (10 μg/mL) treatment. After 48 h, cell apoptosis was measured by flow cytometry analysis (F). Cell viability was evaluated at indicated time points by CCK-8 assay (G). (H) Kaplan-Meier analysis for overall survival and progression free survival in 117 CRC patients according to both IL-33 expression and CD3^+^ T cell density assessed by IHC staining. (I) Representative IHC staining images shows the low and high expression of CD206 in CRC tissues, and scores of CD206 in four groups based on the expression of IL-33 and CD3 were shown as a statistical graph. IL-33^low^CD3^low^ (n = 36), IL-33^low^CD3^high^ (n = 17), IL-33^high^CD3^low^ (n = 21), IL-33^high^CD3^high^ (n = 43). Scale bars, 100 μm. rhIL-33, recombinant human IL-33. ns, no significance. Data are representative of three independent experiments. *P < 0.05, **P < 0.01, ***P < 0.001.

Given the dual roles of IL-33 in both type 1 and type 2 T cell immune responses, we wonder which immune response plays a dominant role in the presence of CRC cells, and whether this response affects the sensitivity of CRC cells to 5-FU. As shown in **Figure 5A**, we designed a culture system consisting of tumor cells alone or a coculture system of tumor cells and CD3^+^ T cells from the PBMCs of HD. Similar with T cells, rhIL-33 stimulation could upregulate ST2 expression in CRC cells (**Figure S6A**). Blocking ST2 decreased the proliferation of CRC cells (**Figure S6B**). Strikingly, either rhIL-33 or IL-33 overexpression alone promoted CRC cell proliferation (**Figure S6C-D**) and suppressed 5-FU-mediated cytotoxicity (**Figure 5B-E**), which was reversed by ST2 blockade, indicating that the role of IL-33 in promoting CRC cell survival mainly relies on an autocrine manner, but not the endogenous nuclear expression. In contrast, 5-FU-mediated cytotoxicity was significantly increased by IL-33 in the presence of CD3^+^ T cells, and was partially attenuated following ST2 blockade (**Figure 5B-E**). Similar results were also obtained in 5-FU-resistant CRC cell lines (**Figure 5F-G**). Overall, these data suggest that IL-33 enhances the sensitivity of CRC cells to 5-FU in a T cell-dependent manner, and that IL-33-mediated T cell responses skew towards the antitumoral Th1-biased axis, but not the Th2 cytokine-associated immune responses.

IL-33 has been found to be positively associated with increased survival and 5-FU sensitivity in patients with CRC (**Figure 1**). Given the controversial role of IL-33 in promoting tumor cell survival and inducing antitumoral T cell responses, we further stratified CRC tissues into four groups according to IL-33 expression and T cell infiltration: IL-33^low^CD3^low^, IL-33^low^CD3^high^, IL-33^high^CD3^low^, and IL-33^high^CD3^high^. Intriguingly, we found that patients with IL-33^high^CD3^high^ showed the best survival, whereas patients with IL-33^high^CD3^low^ showed the worst survival (**Figure 5H**). These results indicate that IL-33 may be biased toward an antitumoral role, such as enhancing 5-FU chemosensitivity, in the TME with abundant T cell infiltration but a protumoral role in the TME with poor T cell infiltration.

Recently, considerable data have suggested a link between IL-33 and M2-like macrophage activation and polarization 28. We also examined the infiltration of M2-like macrophages in patients with CRC with high or low levels of IL-33 expression. The IHC staining results demonstrated that the densities of CD206^+^ M2-like macrophages were higher in patients with IL-33^high^ expression than in those with IL-33^low^ expression, and IL-33^high^ CD3^low^ populations showed the highest densities of CD206^+^ M2-like macrophages (**Figure 5I**). These data suggest that the immunosuppressive M2-like macrophages might have an influence on survival to some extent, particularly in the IL-33^high^ CD3^low^ populations, which had the worst survival.

### IL-33-mediated T cell responses activate both intrinsic apoptotic and immune killing-related signals in CRC cells

**Figure 6:**
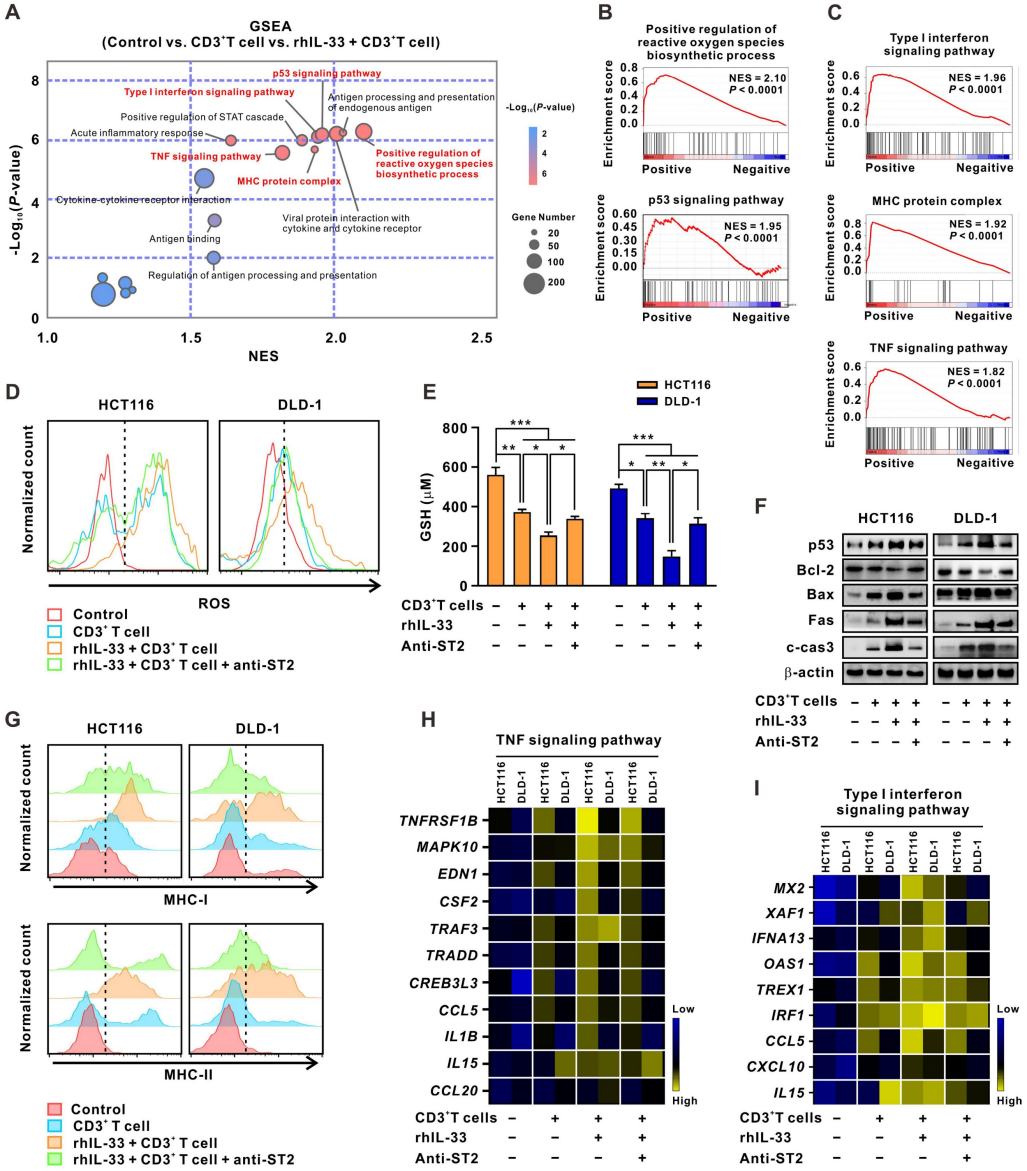
** IL-33-mediated T cell responses activate both intrinsic apoptotic signals and immune killing-related signals of CRC cells.** (A) CD3^+^ T cells were isolated from the PMBCs of 3 CRC patients. CRC cells were alone cultured or cocultured with CD3^+^ T cells treated with or without recombinant human IL-33 (10 ng/mL) for 72 h. GSEA was conducted to identify the significant signaling pathways activated by IL-33-induced T cell responses in CRC cells based on the data of RNA-sequencing. (B-C) GSEA plot showed that the top signaling pathways enriched in CRC cells, including intrinsic apoptotic signals “positive regulation of ROS biosynthetic process” and “p53 signaling pathway” (B) and immune killing-related signals “type I IFN signaling pathway”, “MHC protein complex”, and “TNF signaling pathway” (C). (D-I) CRC cells were alone cultured or cocultured with CD3^+^ T cells pretreated with or without anti-ST2 neutralizing antibodies (1 μg/mL) for 1 h following treatment with or without recombinant human IL-33 (10 ng/mL) for 72 h. The intracellular levels of ROS were detected by flow cytometry (D). The levels of GSH were examined by GSH detection kit (E). Western blot analysis was performed for the expression of p53 signaling-related proteins, including p53, Bcl-2, Bax, Fas, and cleaved-caspase 3 (F). The surface expression of MHC-1 and MHC-II were detected by flow cytometry (G). Heatmaps showing the mRNA expression of TNF signaling- (H) and Type I IFN signaling-related genes (I) determined by qPCR. Data are representative of three independent experiments. *P < 0.05, **P < 0.01, ***P < 0.001.

Thereafter, the molecular mechanisms by which IL-33-mediated T cell responses enhance 5-FU sensitivity in CRC cells were explored. RNA-sequencing analyses were performed on CRC cells alone, CRC cells cocultured with CD3^+^ T cells, or CRC cells cocultured with IL-33-educated CD3^+^ T cells. GSEA analysis of the three groups of CRC cells demonstrated that the tumor cell-intrinsic apoptotic signals “positive regulation of reactive oxygen species (ROS) biosynthetic process” and “p53 signaling pathway,” as well as the immune killing-related signals “type I IFN signaling pathway,” “MHC protein complex,” and “TNF signaling pathway,” were significantly enriched in CRC cells cocultured with IL-33-educated CD3^+^ T cells (**Figure 6A-C**).

As an essential component of the oxidative stress system, ROS has recently been shown to accelerate tumor cell death by inducing apoptosis, necrosis, and autophagy 26. As a result, multiple drugs aiming to increase ROS levels in tumor cells have been gradually included in cancer therapies in clinics 29. ROS and its downstream signaling pathways are dynamically opposed by antioxidants, such as glutathione (GSH), a major intracellular redox molecule that protects cells from oxidative stress 30. Here, we found that ROS levels (**Figure 6D**) were upregulated and GSH levels (**Figure 6E**) were downregulated in CRC cells cocultured with IL-33-educated CD3^+^ T cells. These effects were partially reversed by ST2-neutralizing antibodies. Similar results were obtained by western blot analysis of p53 pathway proteins, including p53, Bcl-2, Bax, and cleaved-caspase 3 (**Figure 6F**).

“Type I IFN signaling pathway,” “MHC protein complex,” and “TNF signaling pathway” are all important components of the T cell-mediated immune killing process 31. The increased expression of MHC-I and MHC-II in CRC cells cocultured with IL-33-educated CD3^+^ T cells was further confirmed by flow cytometry; the expression was partially attenuated by the ST2 blockade (**Figure 6G**). Consistent results were observed by qPCR analysis of the expression of *TNFRSF1B*, *MAPK10*, *EDN1*, *CSF2*, *TRAF3*, *TRADD*, *CREB3L3*, *CCL5*, *IL1B*, *IL15*, and *CCL20* enriched in the “TNF signaling pathway” (**Figure 6H**), as well as *MX2*, *XAF1*, *IFNA13*, *OAS1*, *TREX1*, *IRF4*, *CCL5*, *CXCL10*, and *IL15* enriched in the “type I IFN signaling pathway” (**Figure 6I**). Together, these results indicate that IL-33-mediated T cell responses drive both tumor cell-intrinsic apoptotic and immune killing-related signals in CRC cells. These two kinds of signaling events may converge to reinforce the sensitivity of CRC cells to 5-FU.

### Release of alarmin IL-33 and T cell chemokines by injured CRC cells forms a positive feedback loop to orchestrate antitumoral T cell responses

**Figure 7:**
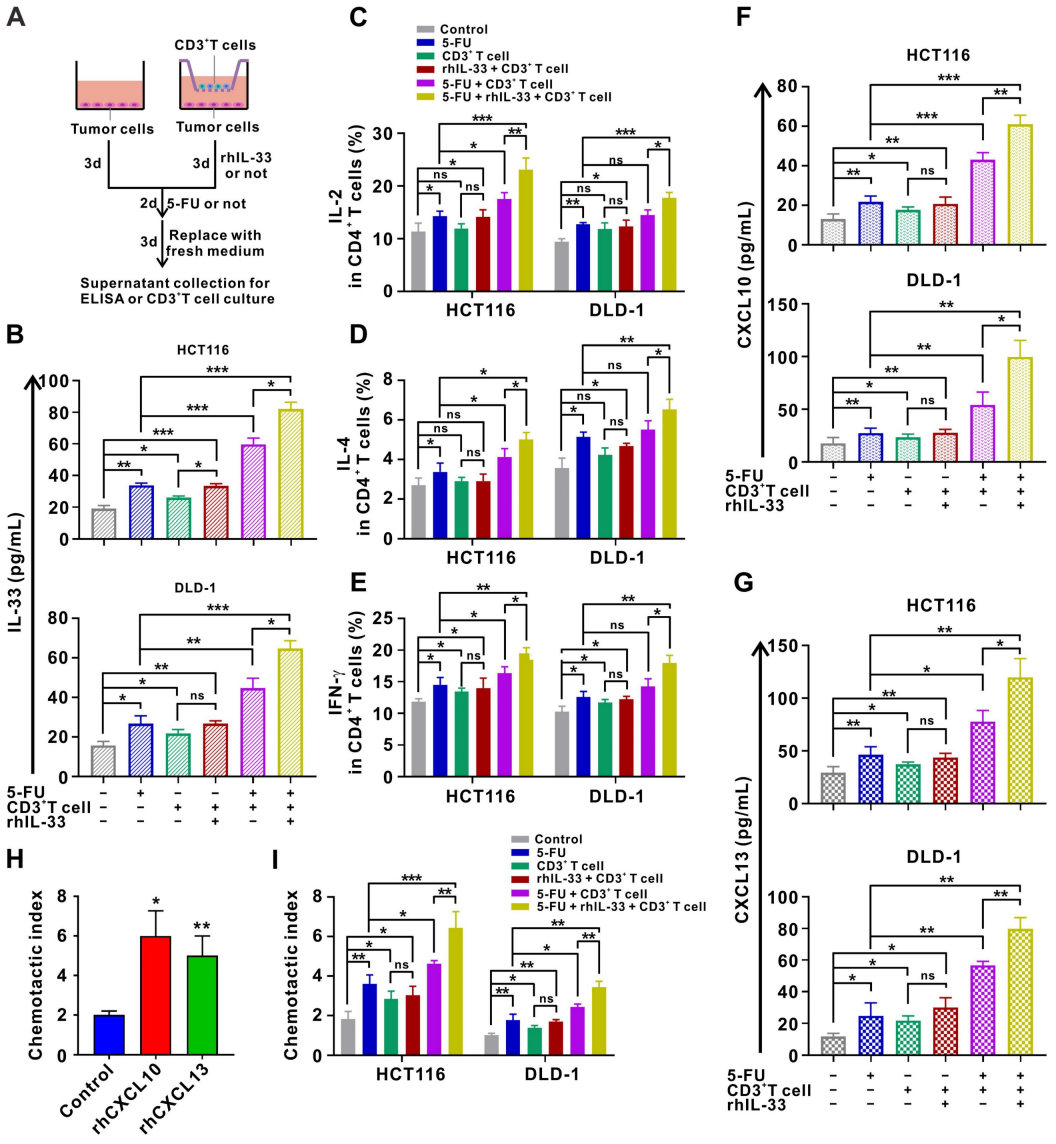
** Injured CRC cells released more IL-33 and T-cell chemokines CXCL10 and CXCL13, forming a positive feedback loop to further augment T cell responses.** (A) Schematic of *in-vitro* treatment. (B-E) CD3^+^ T cells were isolated from the PMBCs of HD. CRC cells were alone cultured or cocultured with CD3^+^ T cells pretreated with or without anti-ST2 neutralizing antibodies (1 μg/mL) for 1 h following treatment with or without rhIL-33 (10 ng/mL) for 72 h, which were then administrated with 5-FU (10 μg/ml). After 48 h, CD3^+^ T cells were removed, and CRC cells in each group were replaced with fresh medium and cultured for 72 h. Indicated supernatants were collected for the detection of IL-33 secretion using ELISA (B) and further treatment of CD3^+^ T cells. The intracellular expression of IL-2 and IL-4 by CD4^+^ T cells and intracellular expression of IFN-γ by CD8^+^ T cells were examined by flow cytometry (C-E). (F, G) Supernatants collected in A were used for the detection of CXCL10 (F) and CXCL13 (G) secretion using ELISA. (H) T-cell migration assay was performed by adding control medium or medium contained rhCXCL10 (10 ng/mL) or rhCXCL13 (10 ng/mL) into lower champers. (I) T-cell migration assay by adding control or indicated medium collected in A into lower champer. rh, recombinant human; ns, no significance. Data are representative of three independent experiments. *P < 0.05, **P < 0.01, ***P < 0.001.

Since IL-33 has been shown to act as an alarmin when released during cell injury 11, we next examined IL-33 expression in CRC cells under different treatment conditions. The results of ELISA demonstrate that IL-33-mediated T cell responses and 5-FU synergistically facilitate IL-33 secretion by CRC cells in the coculture system (**Figure 7A-B**). Moreover, we found that IL-33 released by injured CRC cells in the coculture system further augments the CD4^+^ (Th1 and Th2) and CD8^+^ T cell responses (**Figure 7C-E**), thereby forming a positive feedback loop between T cells and CRC cells.

In particular, IL-33-mediated T cell responses and 5-FU treatment also increased the secretion of the T cell chemokines CXCL10 and CXCL13 by CRC cells in a synergistic manner in the *in vitro* setting (**Figure 7F-G**). Accordingly, secreted CXCL10 and CXCL13 led to increased recruitment of T cells as measured by the chemotaxis assay (**Figure 7H-I**). These findings also support the IHC staining data of clinical samples to some extent, which showed that IL-33 expression was positively correlated with CD3^+^ T cell infiltration in CRC tissues. Overall, we preliminarily surmise that IL-33 may orchestrate an immune-activated TME by mediating a positive feedback loop between T cells and CRC cells, which cooperates with 5-FU to inhibit tumor progression.

### IL-33-mediated T cell responses improve the antitumor activity of 5-FU in mouse models of CRC

**Figure 8:**
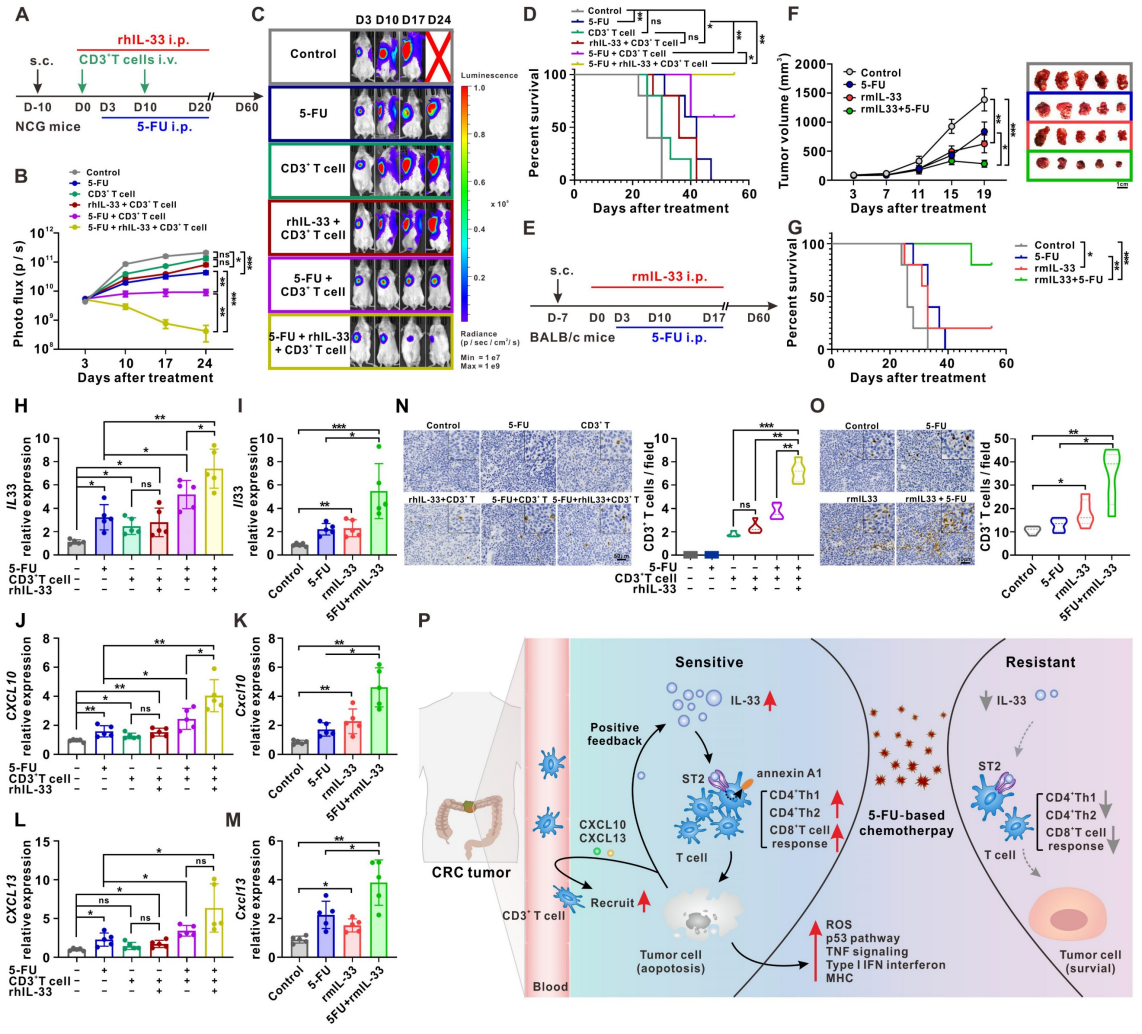
** IL-33-mediated T cell responses improves the antitumor activity of 5-FU in mouse models of CRC.** (A) Experimental scheme for the subcutaneous xenograft model of CRC in NCG mice. Mice are inoculated with 1×10^7^ HCT116 cells subcutaneously and treated with or without rhIL-33 (100 μg/kg intraperitoneal injection, once every other day) from day 0 to 20. CD3^+^ T cells were isolated from PBMCs of HD and infused to mice at day 0 and day 10. 5-FU (10 mg/kg/day intraperitoneal injection) were administrated from days 3 to 20. (B-C) Representative images and photo flux of tumor growth at different time points by using an *in vivo* imaging system. (D) Kaplan-Meier survival curves for the mice in different treatment groups in A. Survival curves were compared using the log-rank test (two-tailed). n = 5 for each group. (E) Experimental scheme for the CT26 colon cancer model in BALB/c mice. Mice are inoculated with 3×10^5^ CT26 cells subcutaneously and treated with or without recombinant murine IL-33 (100 μg/kg intraperitoneal injection, once every other day) from day 0 to 17. 5-FU (10 mg/kg/day intraperitoneal injection) were administrated from days 3 to 17. (F) The tumor volume curves of four different groups and image showing individual tumors excised from different treatment groups at the termination of study. (G) Kaplan-Meier survival curves for the mice in different treatment groups in E. Survival curves were compared using the log-rank test (two-tailed). n = 5 for each group. (H-M) The expression of human *IL-33* (H), *CXCL10* (J), and *CXCL13* (L) in NCG mice-bearing xenografts in A and the expression of mouse *Il33* (I), *Cxcl10* (K), and *Cxcl13* (M) in BALB/c mice-bearing tumors in E were detected by qPCR. (N, O) Representative IHC staining results of CD3 and the number of CD3 positive cells per field from NCG mice-bearing xenografts in A (N) and BALB/c mice -bearing tumors in E (O). (P) Schematic diagram depicting the proposed model of the intricate cellular crosstalk between T cells and tumor cells mediated by IL-33 in the tumor microenvironment of CRC. i.p., intraperitoneal injection; ns, no significance; rm, recombinant murine. Data are representative of three independent experiments. *P < 0.05, **P < 0.01, ***P < 0.001.

To determine whether IL-33-mediated T cell responses affect the chemotherapeutic activity of 5-FU *in vivo*, a subcutaneous xenograft tumor model of CRC was first designed in NCG mice (**Figure 8A**). After 10 days of tumor cell injection, CD3^+^ T cells isolated from the PBMCs of human HD were infused at days 0 and 10 for immune reconstruction. We found that inhibition of tumor growth mediated by the infusion of CD3^+^ T cells was significantly enhanced by rhIL-33 administration, which synergized with 5-FU treatment, resulting in strong tumor regression (**Figure 8B-C**), prolonged survival (**Figure 8D**), and increased expression of the apoptotic marker cleaved-caspase 3 (**Figure S7A**). Owing to the complexity of the original TME, a murine CT26 colon cancer model was established in immunocompetent BALB/c mice to further assess the effect of IL-33 treatment on 5-FU chemotherapy (**Figure 8E**). We found that the additional administration of recombinant murine IL-33 to mice treated with 5-FU led to a more significant delay in tumor growth (**Figure 8F**), prolonged survival (**Figure 8G**), and increased cleaved-caspase 3 (**Figure S7B**) than those observed in control mice or mice receiving either rmIL-33 or 5-FU alone.

We also examined the expression of alarmin IL-33 by CRC cells under the above-mentioned treatment conditions. In line with the *in vitro* results shown in **Figure 7B**, IL-33-mediated T cell responses and 5-FU synergistically facilitated IL-33 expression in tumors from NCG mice (**Figure 8H**) and BALB/c mice (**Figure 8I**). Similar results were obtained with the expression of the T cell chemokines CXCL10 (**Figure 8J-K**) and CXCL13 (**Figure 8L-M**), as well as with the infiltration of T cells (**Figure 8N-O**), in both mouse models. All these data suggest that IL-33 may be a promising adjuvant to generate effective T cell-mediated protective immunity against cancer, with potential applications in CRC treatment through the improved efficacy of 5-FU-based therapies.

Considering the link between IL-33 and M2-like macrophages in other reports 28 and our clinical samples (**Figure 5I**), we examined the intratumoral infiltration of M2-like macrophages in a CT26 colon cancer model that had an immunocompetent TME. The results showed that the additional administration of rmIL-33 increased the infiltration of M2-like macrophages in mice treated with or without 5-FU (**Figure S8**). Despite the increase in the number of immunosuppressive M2-like macrophages, compared with mice that did not receive the treatment, mice treated with rmIL-33 still showed remarkable T cell infiltration (**Figure 8O**), tumor regression (**Figure 8F**), and prolonged survival (**Figure 8G**). Therefore, we infer that the effects of IL-33 on antitumoral T cell responses might be greater than its effects on immunosuppressive M2-like macrophages.

Apart from 5-FU, the release of alarmin IL-33 by CRC cells was also found to increase in injury caused by other chemotherapeutic drugs such as adriamycin and cisplatin (**Figure S9**), indicating that the increase in the released IL-33 by injured CRC is not specific to only injury caused by 5-FU. Therefore, IL-33 may be also a promising adjuvant to other chemotherapy regimens in the treatment of CRC, in addition to 5-FU-based therapies.

## Discussion

5-FU-based chemotherapies have been the main treatment strategy for CRC since the 1950s, but nearly half of the patients with CRC develop drug resistance 3. Recent studies have highlighted the controversial role of IL-33 in tumor development 14-16, 20-22. However, the precise mechanism by which IL-33 participates in CRC regression, as well as in 5-FU resistance, remains unclear. In this study, we first demonstrated that IL-33 expressed by tumor cells was a dominant mediator of antitumoral immunity in 5-FU-sensitive patients. By interacting with its ST2 receptor, IL-33 was shown to trigger the Th1 and Th2 responses of CD4^+^ T cells, as well as the CD8^+^ T cell response, by activating annexin A1-mediated signaling cascades. Mechanistically, IL-33-mediated T cell responses led to the activation of tumor cell-intrinsic apoptotic and immune killing-related signals, thereby enhancing the sensitivity of CRC cells to 5-FU. Moreover, injured CRC cells were found to release more alarmin IL-33 and the T cell chemokines CXCL10 and CXCL13, forming a positive feedback loop to further augment antitumoral T cell responses (**Figure 8P**).

As a multifunctional cytokine, IL-33 has been implicated in the immune regulation of many pathological processes such as immune disorders and various cancers 13-16, 18. In this study, we first identified IL-33 as the dominant mediator of type 1 and type 2 immune responses in a relatively small number of 5-FU-sensitive patients by high-quality bioinformatic analysis of the RNA-sequencing data. We further validated this in enlarged cohorts using multiple analysis methods. However, we did not assess the expression level of IL-33 before and after 5-FU treatment owing to the unavailability of tumor tissues from patients with CRC after 5-FU-based chemotherapy. We also explored the clinical and biological significance of IL-33, described its dual effects on both T cells and tumor cells, and highlighted its bias toward an antitumoral T cell-based axis in the TME of CRC. Our findings are consistent with those reported in recent studies 14-16. For example, IL-33 was found to increase antigen-specific CD8^+^ T cell responses and elicit effector-memory CD8^+^ T cells in a human papillomavirus-associated tumor model; it was therefore considered to be an immunoadjuvant in vaccinations against pathogens, including in antitumor immunotherapy 15. Upregulation of IL-33 has previously been shown to activate CD8^+^ T cells and natural killer cells as well as inhibit tumor growth and metastasis in mouse models of melanoma and lung cancer 14, 16. In addition, alarmin IL-33 was also shown to mediate protective CD8^+^ T cell responses in antiviral immunity 17. Th2 cytokines have been implicated in blocking tumor growth in previous studies on tumor immunotherapy. Indeed, the role of IL-33 in promoting a Th2 response in the pathogenesis of asthma and activating mast cells in the mediation of joint inflammation, atopic dermatitis, and anaphylaxis is well established 13. Kwon* et al.* demonstrated that Th2 cytokines might create an unfavorable TME for tumor growth independent of adaptive immunity and found that local production of IL-33 established a high number of type 2 innate lymphoid cells with potent antitumor activity 32. Therefore, in the present study, we cannot exclude the possibility that the IL-33-mediated T cell response might inhibit CRC progression via Th2 cytokines. All data provide strong evidence that IL-33 is a potent immune regulator that activates CD4^+^ (Th1 and Th2) and CD8^+^ T cells. Thus, IL-33 may be a promising novel cytokine for tumor immunotherapy through its promotion of cancer-eradicating T cell immune responses.

In contrast, IL-33 has been shown to enhance the malignancy of tumor cells via distinct mechanisms 20, 21. Consistently, our findings showed that IL-33 promoted tumor cell proliferation and prevented chemotherapy-induced apoptosis in an autocrine manner. We mainly focused on the role of secreted IL-33 by CRC cells in this study. The endogenous nuclear expression of IL-33 was also reported as a chromatin-associated nuclear factor in other studies 12, and we will further explore the biological function of endogenous nuclear IL-33 in future studies. Previously, IL-33 was found to increase tumor cell migration, invasion, and proliferation through regulation of the ERK and JNK signaling pathways in ovarian cancer 20. In renal cell carcinoma, IL-33 was shown to stimulate tumor cell proliferation and chemoresistance 21. Strikingly, we found a significant correlation between IL-33, enhanced sensitivity to 5-FU chemotherapy, and increased survival in patients with CRC. Furthermore, the stimulatory effects of an IL-33-mediated immune response on tumor cell apoptosis far exceeded the inhibitory effect of IL-33 itself on tumor cell apoptosis during 5-FU chemotherapy. Activation of tumor cell-intrinsic and immune killing-related apoptotic signals in CRC cells will enhance the sensitivity of CRC cells to 5-FU, eventually leading to tumor regression. In the current study, we clearly elucidated the distinct mechanisms underlying the dual role of IL-33 in tumor development. Furthermore, we propose that IL-33 is biased toward an antitumoral role of enhancing 5-FU chemosensitivity by modifying the TME through CD4^+^ and CD8^+^ T cells. Clinically, the best survival rates were found in patients with IL-33^high^CD3^high^, whereas patients with IL-33^high^CD3^low^ had the worst survival rates. These findings suggest that IL-33 may exert its antitumoral effect in a TME consisting of abundant, and not poor, T cell infiltration. Thus, evaluating the levels of T cell infiltration may be necessary when using IL-33 adjuvant therapy, as patients with high-level T cell infiltration may be more sensitive to 5-FU-based therapies.

Recent studies have reported a link between IL-33 and M2-like macrophage polarization 28. IL-33-induced metabolic reprogramming controls the differentiation of alternatively activated macrophages and the resolution of inflammation 28. Consistent with the findings of these studies, we found that densities of CD206^+^ M2-like macrophages in the clinical samples of CRC tissues were higher in IL-33^high^ populations, with the highest densities in the IL-33^high^ CD3^low^ populations which had the worst survival. Thus, we could not exclude the influence of M2-like macrophages on patient survival. Although IL-33 administration increased the infiltration of M2-like macrophages in the CT26 colon cancer model in the present study, it led to a significant increase in T cell infiltration and tumor regression. Therefore, we preliminarily inferred that the effects of IL-33 on antitumoral T cell response might exceed its effects on immunosuppressive M2-like macrophages, resulting in a potent antitumor effect in CRC. The difference between CD3 populations with IL-33^high^ is interesting; it might be attributed to the association between macrophages and T cell apoptosis via various mechanisms including synaptic interactions 33, Fas-FasL signaling 34, and the immunosuppressive PD-1-PD-L1 axis 35. The crosstalk between IL-33-mediated T cell response and M2-like macrophages will be further investigated in future studies. Naturally, we did not exclude other immune cells as targets of IL-33. Some studies have reported the IL-33-ST2 axis as an endogenous danger signal that affects various types of cells, including macrophages, mast cells, basophils, eosinophils, and specific subsets of innate lymphoid cells, in the TME 23, 24. Immune profiling analysis revealed that apart from T cells, higher expression levels of “dendritic cells resting” and lower expression levels of “mast cell activated” and “neutrophils” were also observed in 5-FU-sensitive tissues (**Figure S1D**), which favors our future studies on these immune cells. In this study, *in vivo* subcutaneous tumor implantation models were established in both immunodeficient and immunocompetent mice. We are currently investigating additional models of CRC including spontaneous, orthotopic, and syngeneic models. The tumor-promoting role of IL-33 in APC mice might be attributed to the heterogeneity of the TME among different mouse models 36, which will also be further explored.

Besides, IL-33 was reported to have full-length (33 Kd) and mature (26 Kd) forms, and the full-length protein can be released from cells and signal via the IL1RL1/ST2 receptor 12. We found that IL-33 mainly functions as a secreted protein in CRC cells or during the crosstalk of CRC cells with T cells, and that the IL-33 protein expressed by CRC cells showed a single band of approximately 33 Kd in the western blot analysis. Therefore, IL-33 likely functions as a full-length form in the CRC model in this study.

We found that annexin A1 was essential for IL-33-induced CD4^+^ (Th1 and Th2) and CD8^+^ T cell responses. Indeed, annexin A1 has been identified as an endogenous inflammatory factor that alters the TME and tumor immune response 37. More specifically, annexin A1 acts as a molecular “tuner” of T cell receptor signaling by increasing the activation of the nuclear factor of activated T cells and activator protein-1 and modulating T cell activation and differentiation, eventually contributing to increased skewing toward Th1 cells 38. However, we only confirmed the role of annexin A1 in IL-33-induced T cell responses by using T cells isolated from PBMCs owing to the accessibility and *in vitro* longevity of TILs isolated from patients with CRC. An increased Th2 lineage commitment was observed in annexin A1-deficient T cells in a mouse model of allergic inflammation, which is inconsistent with our findings and warrants further validation in tumor models 39. A broader role for annexin A1 beyond inflammation has been reported, including in tumor cell proliferation, differentiation, apoptosis, invasion, angiogenesis, and metastasis 40. For example, in breast cancer cells, the ANXA1/G protein-coupled receptor FPR2 was found to facilitate tumor cell proliferation by activating the PI3K/AKT/p70S6K/cyclin D1 axis 41. Based on these previous studies and our data showing that *IL-33* is significantly associated with *ANXA1* in human CRC samples, upregulates annexin A1 expression in T cells, and increases tumor cell proliferation, we propose that annexin A1 might play a role in IL-33-enhanced proliferation of CRC cells. Further studies are required to determine whether this is the case.

## Conclusions

Our results identified a direct and functional connection between IL-33 and 5-FU sensitivity in CRC, highlighted the involvement of antitumoral T cell responses in this process through a signaling cascade activated by annexin A1, and unveiled the detailed molecular mechanisms underlying the enhanced 5-FU sensitivity of CRC cells. These findings support future studies on IL-33 as a potential predictive biomarker for 5-FU chemosensitivity and favorable prognosis, as well as a promising adjuvant to generate effective T-mediated protective immunity against cancer, thereby improving the therapeutic efficacy of 5-FU-based regimens in the treatment of CRC.

## Supplementary Material

Supplementary figures and methods.

## Figures and Tables

**Table 1:** Univariate and multivariate analysis of overall survival and progression-free survival in patients with colorectal cancer.

Variable	Overall survival				Progression free survival			
	Univariate cox		Multivariate cox		Univariate cox		Multivariate cox	
	*P*-value	HR (95%CI)	*P*-value	HR (95%CI)	*P*-value	HR (95%CI)	*P*-value	HR (95%CI)
**Age**								
>= 60 years or < 60 years	0.738	1.111 (0.666-2.055)			0.370	0.761 (0.418-1.383)		
**Gender**								
Male or female	0.323	1.404 (0.716-2.751)			0.463	1.269 (0.673-2.393)		
**Site of lesion**								
Rectum or colon	0.404	0.742 (0.368-1.492)			0.072	0.520 (0.255-1.060)		
**Histological differentiation**								
Poor or well	0.332	0.724 (0.377-1.390)			0.721	1.121 (0.598-2.102)		
**Tumor size**								
>= 5cm or < 5cm	0.906	0.962 (0.510-1.814)			0.624	1.161 (0.639-2.107)		
**Pathological type**								
Adenocarcinoma or others	0.019	0.085 (0.011-0.673)	0.024	0.087 (0.011-0.720)	0.026	0.096 (0.012-0.750)	0.033	0.103 (0.013-0.833)
**TNM stage**								
IV or I-III	0.007	2.407 (1.275-4.544)	0.010	2.356 (1.231-4.511)	0.001	2.676 (1.467-4.882)	0.004	2.489 (1.348-4.597)
**CEA**								
High or normal	0.588	1.185 (0.642-2.188)			0.562	1.190 (0.661-2.139)		
**CA19-9**								
High or normal	0.570	1.242 (0.587-2.628)			0.424	1.337 (0.656-2.721)		
**IL-33 expression**								
High or low	0.007	0.413 (0.218-0.781)	0.018	0.459 (0.241-0.874)	0.0001	0.315 (0.168-0.587)	0.001	0.355 (0.189-0.668)

## Abbreviations

FU: 5-fluorouracil
CRC: colorectal cancer
DEGs: differentially expressed genes
ELISA: enzyme linked immunosorbent assay
GO: Gene Ontology
GSEA: gene-set enrichment analysis
GSH: glutathione
IHC: immunohistochemical
IL: interleukin
MAPK: mitogen-activated protein kinase
PBMCs: peripheral blood mononuclear cells
qPCR: quantitative polymerase chain reaction
rh: recombinant human
rm: recombinant murine
ROS: reactive oxygen species
siRNAs: short interfering RNAs
ST2: suppression of tumorigenicity 2
Th: T helper
TIF: tumor interstitial fluid
TILs: tumor-infiltrating lymphocytes
TME: tumor microenvironment
