# Supplementary material for: Alarmin IL-33 orchestrates antitumoral T cell responses to enhance sensitivity to 5-fluorouracil in colorectal cancer
Source: Theranostics. 2023 Mar 13;13(5):1649–68. doi: 10.7150/thno.80483 (PMC10086207; doi:10.7150/thno.80483)
Supplement: Supplementary file 1 — Supplementary figures and methods. [file thnov13p1649s1.pdf]

Supplementary materials for

Alarmin IL-33 orchestrates antitumoral T cell responses to enhance sensitivity to 5-Fluorouracil in colorectal cancer

Mengjia Song[1,2#], Jieying Yang[1,3#], Muping Di[1,3#], Ye Hong[1,2#], Qiuzhong Pan[1,3], Yufei Du[1,3], Tong Xiang[1,3], Juan Liu[1,2,4], Yan Tang[1,3], Qijing Wang[1,3], Yongqiang Li[1,3], Jia He[1,3], Hao Chen[1,3], Jingjing Zhao[1,3], Desheng Weng[1,3], Yizhuo Zhang[1,2*] and Jian-Chuan Xia[1,3*]

[1]Collaborative Innovation Center for Cancer Medicine, State Key Laboratory of Oncology in South China, Sun Yat-sen University Cancer Center, Guangzhou, China. [2]Department of Pediatric Oncology, Sun Yat-sen University Cancer Center, Guangzhou, China. [3]Department of Biotherapy, Sun Yat-sen University Cancer Center, Guangzhou, China. [4]Department of Pediatric Oncology, The Fifth Affiliated Hospital, Guangzhou Medical University, Guangzhou, China.

This PDF file includes: Figure S1, Figure S2, Figure S3, Figure S4, Figure S5, Figure S6, Figure S7, Figure S8, Figure S9, and Supplementary Methods.

Figures and Figure Legends

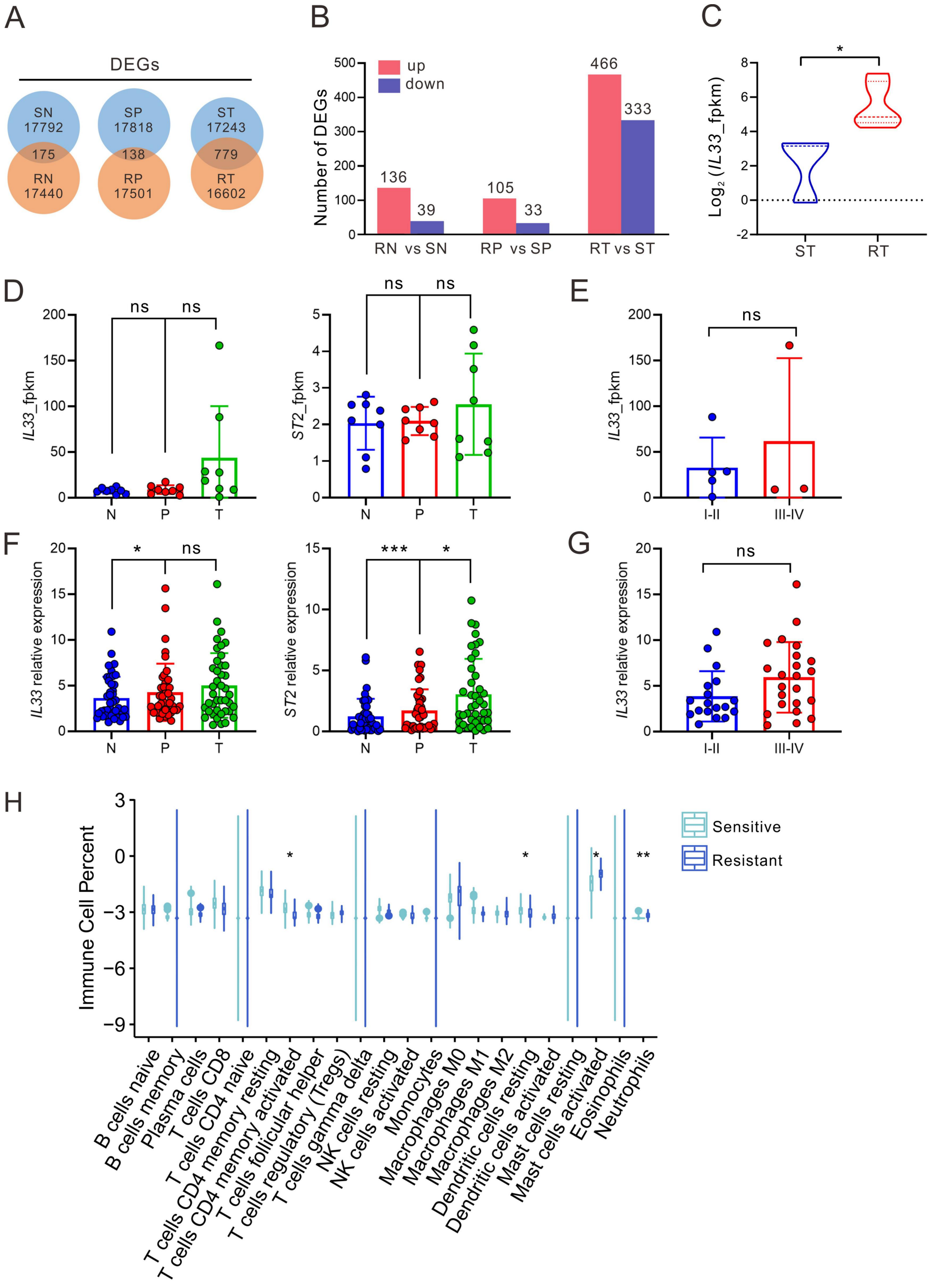

Figure S1. (A) Number of DEGs among SN versus RN, SP versus RP, and ST versus RT based on RNA-sequencing data of tissues from CRC patients. DEG, differentially expressed gene. SN: paired normal tissues from 5-FU sensitive patients. SP: paired

peritumor tissues from 5-FU sensitive patients. ST: tumor tissues from 5-FU sensitive patients. RN: paired normal tissues from 5-FU resistant patients. RP: paired peritumor tissues from 5-FU resistant patients. RT: tumor tissues from 5-FU resistant patients. (B) Number of up-regulated and down-regulated DEGs in A. (C) The fpkm of *IL33* in 5-FU-sensitive and -resistant tumor tissues based on RNA sequencing were shown as a statistical graph by showing Log2 values. (D) The fpkm of *IL33* and *ST2* in normal, peritumor, and tumor tissues based on RNA sequencing. N: normal; P: peritumor; T: tumor. (E) The fpkm of *IL33* in tumor tissues from different clinical stages of CRC patients. (F) The expression of *IL33* and *ST2* in 124 matched normal, peritumor, and tumor tissues form 41 CRC patients were examined by qPCR. N: normal; P: peritumor; T: tumor. (G) *IL33* expression in 41 tumor tissues from different clinical stages of CRC patients, including 18 cases with I-II stages and 23 cases with III-IV stages. (H) The algorithm "CIBERSORT" was used to quantify 22 kinds of immune cells fractions in 5-FU-sensitive and -resistant tissues based on the RNA-sequencing data. The Wilcoxon-Mann-Whitney test was used to compare non-normal distribution continuous variables between two groups. ST: sensitive tumor, RT: resistant tumor. ns, no significance. Data are representative of three independent experiments. *$P < 0.05$, **$P < 0.01$.

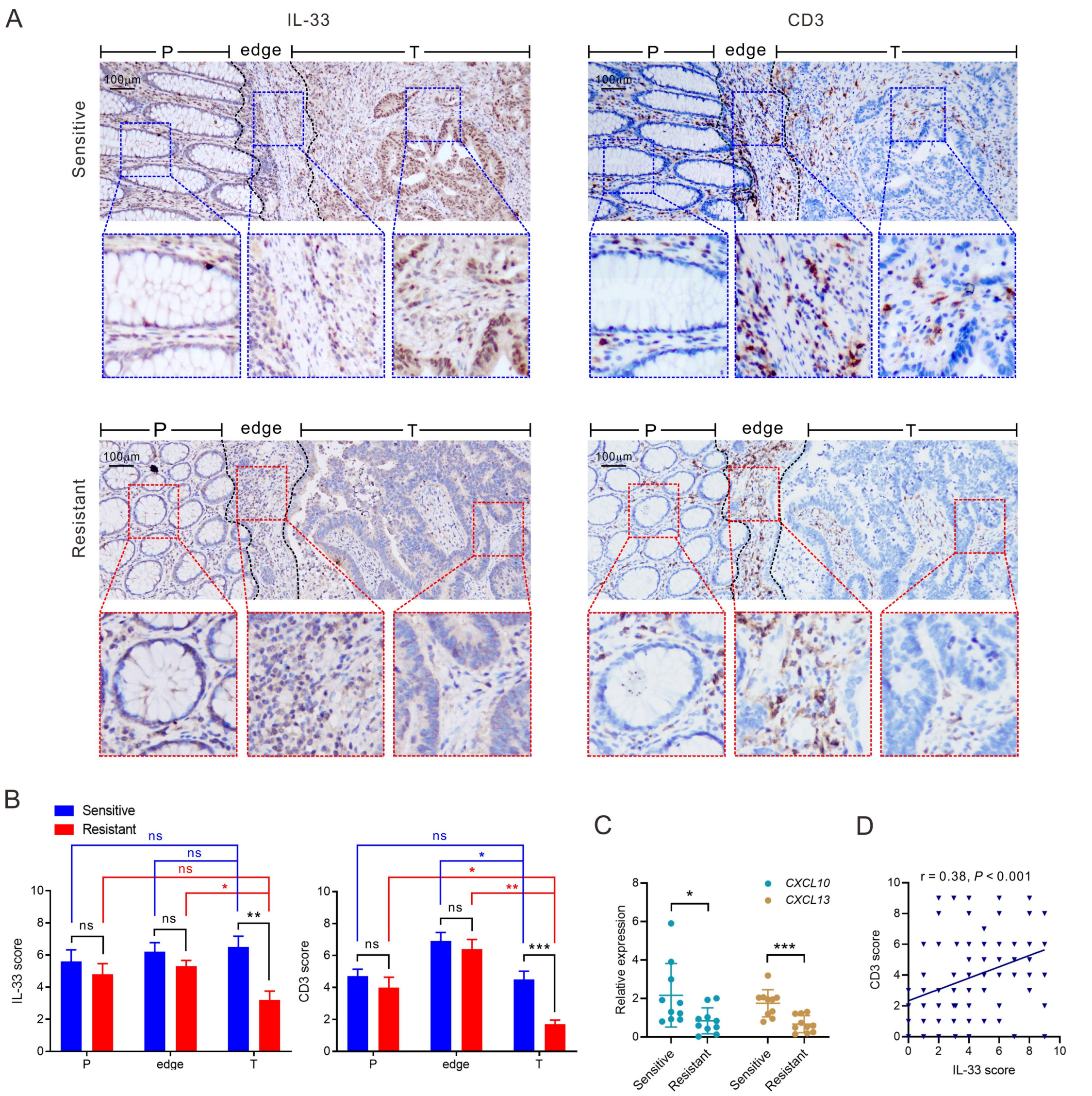

Figure S2. IL-33 expression level is positively correlated CD3+ T cell density in CRC tissues. (A–B) Consecutive sections of 5-FU-sensitive and -resistant tissues from CRC patients were used to analyze the expression IL-33 and CD3 by IHC staining. Representative micrographs and scores for IHC staining of IL-33 and CD3 in the peritumoral (P), edge, and tumoral (T) regions of CRC tissues (n = 10). Scale bars, 100 μm. (C) The mRNA expression *CXCL10* and *CXCL13* in tumor tissues from 5-FU-sensitive (n = 10) and resistant (n = 10) CRC patients was detected by qPCR. (D) Correlation between IL-33 and CD3 expression in the tumoral regions of 117 CRC tissues based on the IHC staining was analyzed by Pearson's correlation method. ns, no significance. Data are representative of three independent experiments. $*P < 0.05$, $**P < 0.01$.

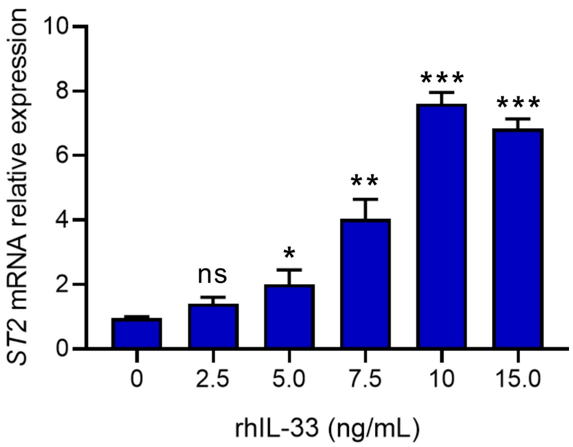

Figure S3. IL-33 upregulates *ST2* expression of $CD3^+$ T cells in a dose-dependent manner. $CD3^+$ T cells isolated from the PMBC of healthy donors were activated with CD3/CD28 T cell Activator for 48 h and then treated with different doses of recombinant human IL-33 for 72 h. The expression of *ST2* by $CD3^+$ T cells was examined by qPCR. Data are representative of three independent experiments. $^*P < 0.05$, $^{**}P < 0.01$, $^{***}P < 0.001$.

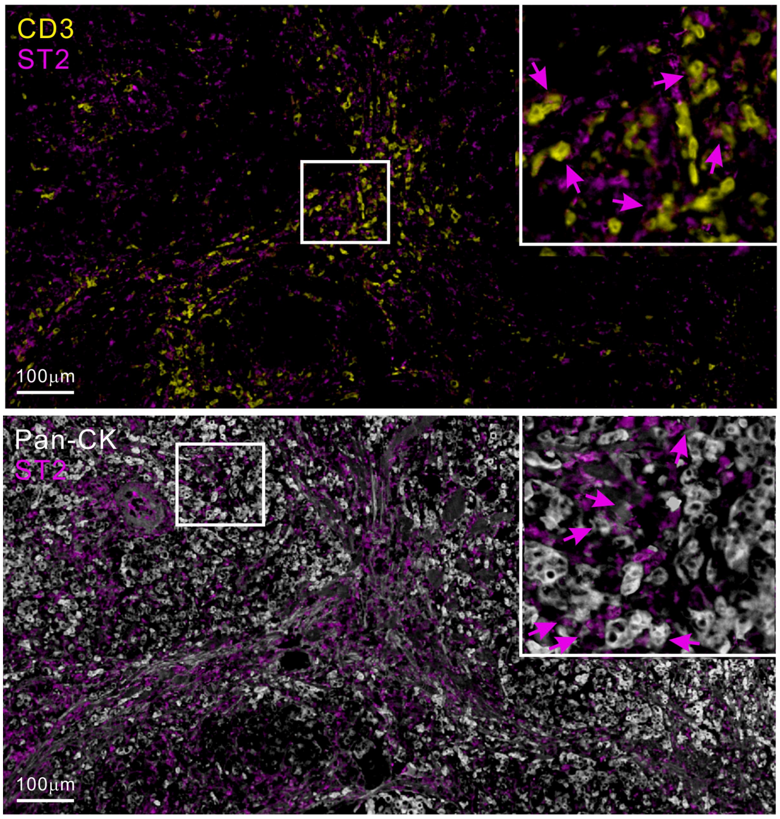

Figure S4. Multiplex immunofluorescence staining for the co-expression pattern of ST2 (magenta) with CD3 (yellow) and Pan-CK (white) in CRC tissues. Representative cells are indicated by arrows, including $ST2^{+}$ $CD3^{+}$cells (magenta, top) and $ST2^{+}$Pan-CK^{+}cells (magenta, bottom). Figure panel pairs the representative images taken with different zooming options. Scale bar, 100μm. Pan-CK, Pan-Cytokeratin.

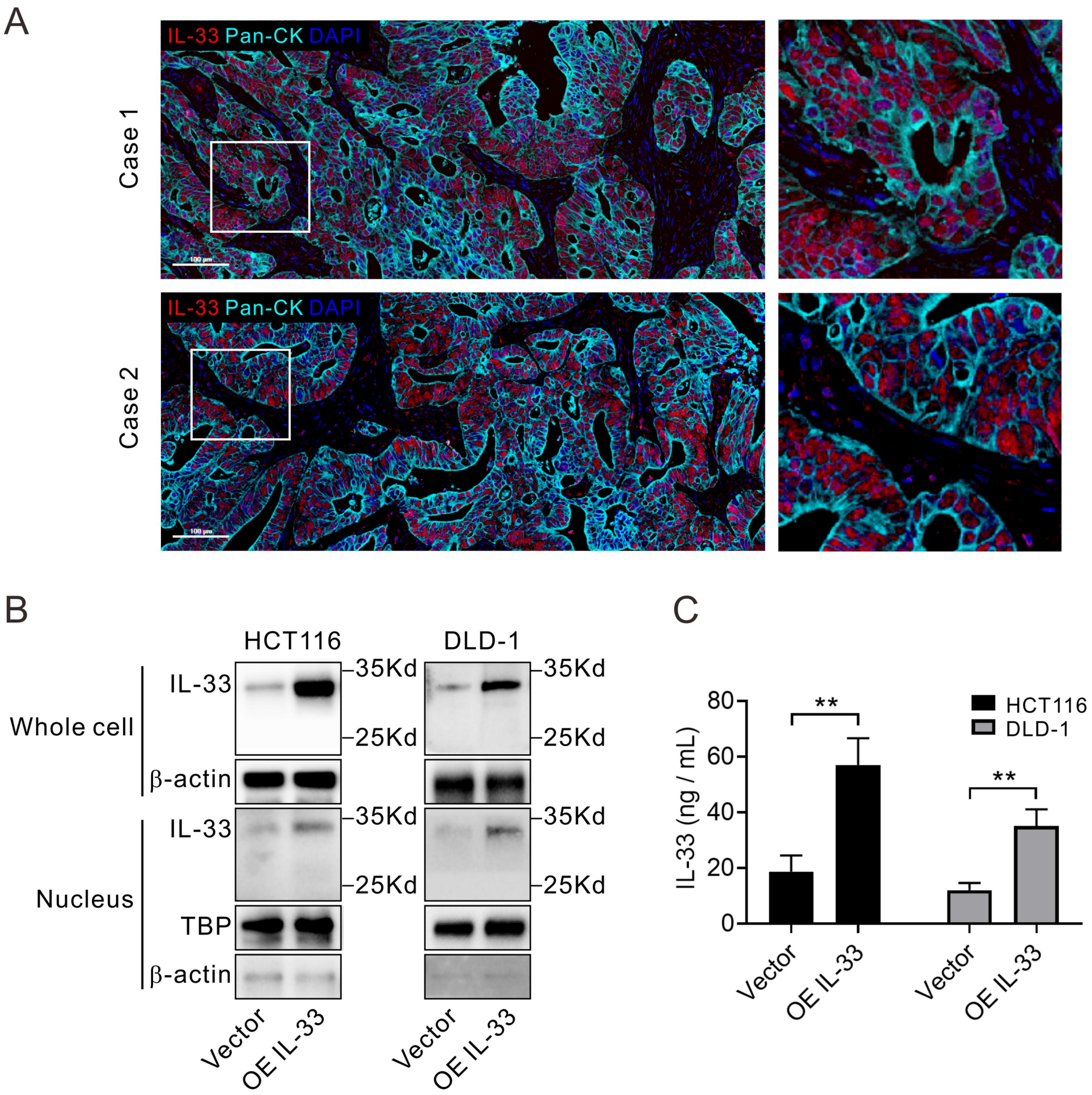

Figure S5. IL-33 was mainly derived from tumor cells in CRC tissues. (A) The co-expression pattern of IL-33 (red), Pan-CK (cyan), and DAPI (blue) by multiplex immunofluorescence staining. Figure panel pairs the representative images taken with different zooming options. Scale bar, 100 μm. Pan-CK, Pan-Cytokeratin. (B) The whole cell and nucleus proteins are extracted from CRC cells with or without IL-33 overexpression, and western blotting analysis shows the expression of IL-33 in the whole cell and nucleus. TBP is used as the control of nucleus proteins. (C) The secretion of IL-33 was detected in the supernatants of CRC cells with or without IL-33 overexpression. Data are representative of three independent experiments. $^{**}P < 0.01$.

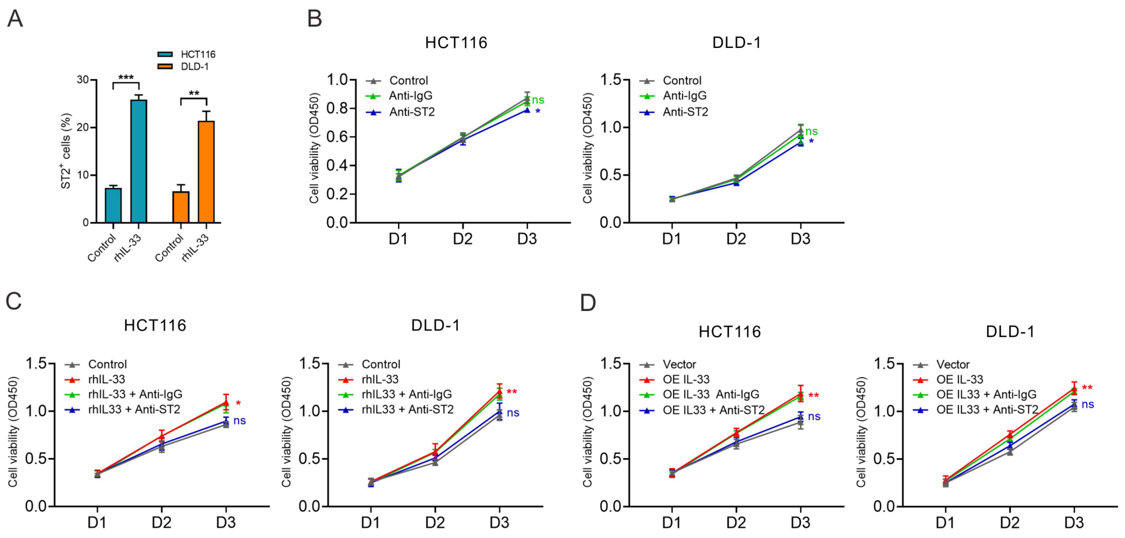

Figure S6. IL-33 promotes CRC cell proliferation in an autocrine manner. (A) CRC cells were treated with or without rhIL-33 (10 ng/mL) for 72 h. The surface expression of ST2 were examined by flow cytometry. (B–D) CRC cells were pretreated with anti-IgG or anti-ST2 neutralizing antibodies (1 μg/mL) for 1 h (B); CRC cells were pretreated with anti-IgG or anti-ST2 neutralizing antibodies (1 μg/mL) for 1 h (B), followed by recombinant human IL-33 (10 ng/mL) treatment (C); IL-33 overexpressing CRC cells were pretreated with anti-IgG or anti-ST2 neutralizing antibodies (1 μg/mL) for 1 h (D). Cell viability was evaluated at indicated time points by CCK-8 assay. Data are representative of three independent experiments. **P* < 0.05, ***P* < 0.01.

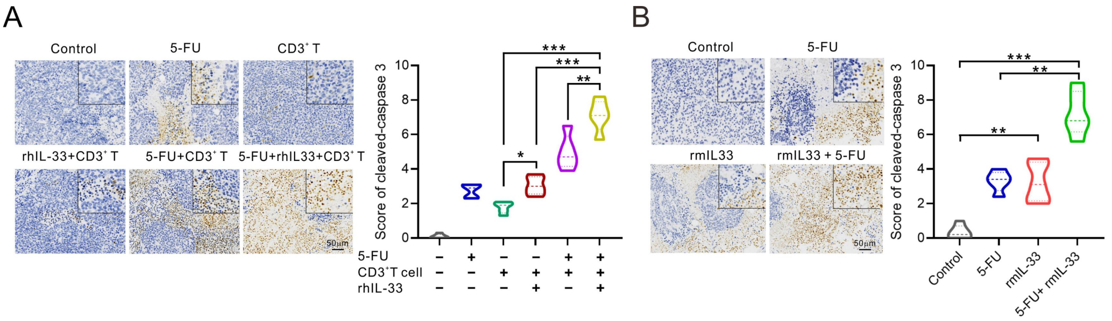

Figure S7. IL-33-mediated T cell responses and 5-FU synergistically increase the expression of cleaved-caspase 3 *in vivo*. (A, B) IHC staining shows the expression of cleaved-caspase 3 in the xenografts from NCG mice (A) and in the tumors from BALB/c mice of the CT26 colon cancer model (B). The scores of CD206 were shown as a statistical graph. n = 5 for each group. Scale bars, 50 μm. Data are representative of three independent experiments. *$P < 0.05$, **$P < 0.01$, ***$P < 0.001$.

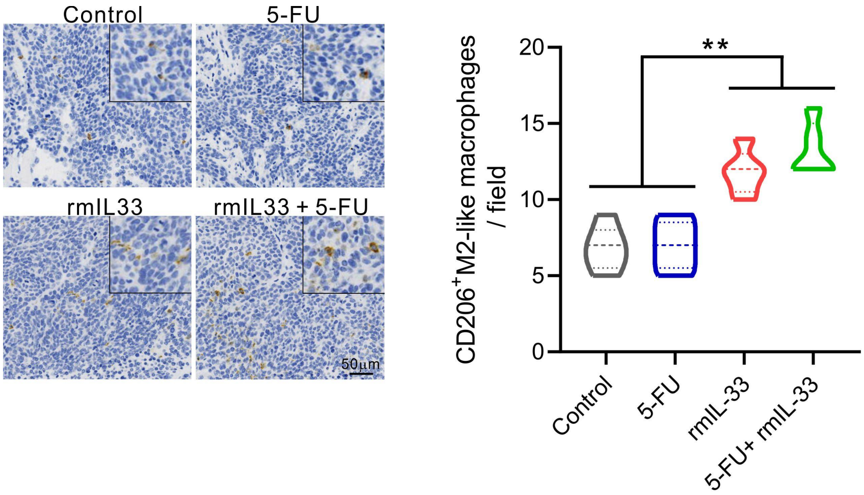

Figure S8. The expression of CD206 in tumors from the CT26 colon cancer model. Representative IHC staining results of CD206. The number of CD206 positive cells per field in four different groups of tumors from BALB/c mice was shown as a statistical graph. n = 5 for each group. Scale bars, 50 μm. Data are representative of three independent experiments. ***$P < 0.01$.

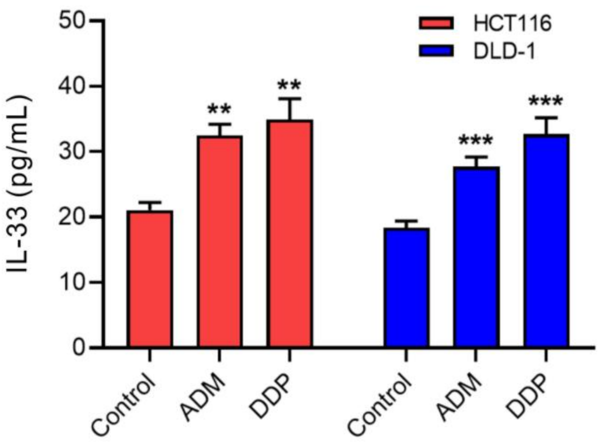

Figure S9. Release of alarmin IL-33 could be induced by other chemotherapeutic drugs. HCT116 cells and DLD-1 cells were treated with ADM (50 nmol/L) or DDP (1 μg/mL) for 72 h, respectively. Supernatants were collected for the detection of IL-33 secretion using ELISA. ADM, adriamycin; DDP, cisplatin. Data are representative of three independent experiments. $^{**}P < 0.01$, $^{***}P < 0.001$.

Supplementary Methods

CRC cell lines and cell culture

Human CRC cell lines, HCT116 and DLD-1, and mouse colon cancer cell line, CT26 were purchased from the American Type Culture Collection (Manassas, VA, USA). All CRC cells were used within 15 passages and had been confirmed without mycoplasma contamination. Cells were cultured in DMEM (Gibco, Invitrogen Carlsbad, CA, USA) supplemented with 10% fetal bovine serum (Gibco), 100 U/ml penicillin and 100 U/ml streptomycin in 5% CO_2 at 37 °C.

Immunohistochemistry and evaluation

The IHC staining protocol was described elsewhere [1]. The following anti-human primary antibodies were used: IL-33 (Abcam, Cambridge, MA, USA, 1:500), CD3 (Abcam, 1:1000), CD206 (Abcam, 1:1000) and cleaved-caspase 7 (Cell Signaling Technology, Boston, MA, USA, 1:2000). The intensity of IHC staining was assessed using the following criteria: 0, none; 1, light yellow; 2, dark yellow; and 3, brown. The extent of IHC staining was scored as 0, > 0 and <= 3% stained; 1, > 3% and <= 5% stained; 2, > 5% and <= 10% stained; and 3, >10% stained. Five random fields per sample were evaluated using a light microscope (Olympus, Tokyo, Japan). The final scores were calculated by multiplying the intensity scores by the extent scores. Samples were divided into four expression grades according to the IHC score as follows: 0, −, negative; > 0 and <= 3, +, weak; > 3 and <= 6, ++, medium; and > 6 and <= 9, strong, +++. Grades "-" and "+" were identified as "low expression", and "++ and +++" were identified as "high expression". All scoring was performed by a pathologist blinded to the clinical outcomes.

Immunofluorescence staining

Tissue multiplex immunofluorescence staining was performed using a Pano-Panel Kit according to the manufacturer's instructions (Panovue, Beijing, China). Images were batch-scanned and analyzed using the Vectra Polaris Automated Quantitative Pathology Imaging System (Perkin Elmer, Waltham, MA, USA) and HALO Image

Analysis Platform (lndica Labs, Albuquerque, USA), respectively. The following rabbit anti-human primary antibodies were used for multiplex immunofluorescence staining: CD4 (Abcam, 1:500), GATA3 (Abcam, 1:500), T-bet (Abcam, 1:300), IL-33 (Abcam, 1:500), Pan-Cytokeratin (Abcam, 1:400), and ST2 (R&D Systems, UAS, 1:300). For cell immunocytochemistry staining, cells were fixed in 4% paraformaldehyde for 15 min at room temperature, washed with PBS and permeabilized with or without 0.1% Triton X-100 in PBS for 10 min. Then, cells were incubated with rabbit anti-human Annexin A1 primary antibody (Abcam, 1:100) at 4 °C overnight. After washing with PBS, the cells were incubated with an appropriate fluorescent dye-conjugated secondary antibody for 1 h, and then nuclear staining with DAPI for 10 min. Images were obtained by laser scanning confocal microscopy (LSM880, Zeiss).

Bioinformatics analysis

The Cancer Genome Atlas dataset for CRC was obtained from the UCSC Cancer Browser (https://genome-cancer.ucsc.edu). Gene expression levels based on the mRNA sequencing data of The Cancer Genome Atlas are shown as the mean ± standard error of the mean of triplicate determinations. Gene-set enrichment analysis was performed using GSEA 2.0.9 (http://www.broadinstitute.org/gsea/) according to The Cancer Genome Atlas mRNA sequencing data or RNA-sequencing data of CRC tissues and cells.

Co-culture of $CD3^+$ T cells with CRC cells

For the co-culture experiments involving $CD3^+$ T cells and CRC cell lines, $CD3^+$ T cells isolated from the PMBCs of healthy donors and activated with 25 μg/mL CD3/CD28 T cell Activator (STEMCELL) and 50 U/mL rhIL-2 for 48 h.

To examine the role of CRC cell-derived IL-33 in T cell responses, $CD3^+$ T cells were pretreated with anti-IgG or 1 μg/mL anti-ST2 neutralizing antibodies (R&D Systems) for 1 h, respectively, following by coculture with CRC cells with or without IL-33 overexpression for 72 h in 24-well Transwell system. Then $CD3^+$ T cells were

harvested for flow cytometry and CD4$^+$ T sorting using MACS magnetic sorting system for western blotting.

To evaluate the effects of IL-33-induced T response on the sensitivity of CRC cells to 5-FU, wild type or 5-FU resistant CRC cells and CD3$^+$ T cells were pretreated with anti-IgG or 1μg/mL anti-ST2 neutralizing antibodies (R&D Systems) for 1 h, respectively. Then, CRC cells were alone cultured or cocultured with CD3$^+$ T cells with or without rhIL-33 (10 ng/mL, Peprotech) treatment, followed by 5-FU (10 μg/mL, Sigma-Aldrich) treatment in Transwell system. After 48 h, cell apoptosis was measured by flow cytometry analysis. Cell viability was evaluated at indicated time points by CCK-8 assay; vector or IL-33-overexpressing CRC cells and CD3$^+$ T cells were pretreated with anti-IgG or 1 μg/mL anti-ST2 neutralizing antibodies (R&D Systems) for 1 h, respectively. Then, CRC cells were alone cultured or cocultured with CD3$^+$ T cells, followed by 5-FU (10 μg/mL, Sigma-Aldrich) treatment in Transwell system. After 48 h, cell apoptosis was measured by flow cytometry analysis. Cell viability was evaluated at indicated time points by CCK-8 assay.

To examine the influence of IL-33-mediated T cell responses and 5-FU treatment on the secretion of IL-33, CXCL10, and CXCL13 by CRC cells, as well as the influence on the feedback on T cell response, CRC cells were alone cultured or cocultured with CD3$^+$ T cells pretreated with or without anti-ST2 neutralizing antibodies (1 μg/mL) for 1 h following treatment with or without rhIL-33 (10 ng/mL, peprotech) for 72 h, which were then administrated with 5-FU (10 μg/mL, Sigma-Aldrich). After 48 h, CD3$^+$ T cells were removed, and CRC cells in each group were replaced with fresh medium and cultured for 72 h. Indicated supernatants were collected for the detection of the secretion of IL-33, CXCL10, and CXCL13 using ELISA and further treatment of CD3$^+$ T cells for 72 h. Then CD3$^+$ T cells were harvested for flow cytometry.

Total RNA extraction and quantitative RT-PCR (qPCR)

Total RNA was obtained from tissues or cells by using the TRIzol reagent (Invitrogen Corporation) according to the manufacturer’s instructions. The concentration and

purity of the RNA were assessed by using a NanoDrop 2000 instrument (Thermo Scientific, Waltham, MA, USA). First-strand cDNA was synthesized from total RNA by using a GoScript Reverse Transcription System (Promega, Madison, WI, USA). RT-PCR was performed using GoTaq qPCR Master Mix (Promega). *GAPDH* was used as an endogenous control for normalization.

Western blot analysis

The western blot protocol was described elsewhere [2]. Nucleus proteins of CRC cells were extracted using a Nuclear and Cytoplasmic Protein Extraction Kit (Beyotime Biotechnology, Shanghai, China) according to the manufacturer's instructions. TBP was used as an control for nucleus proteins. Primary anti-human antibodies used were as follows: STAT1 (Cell Signaling Technology, 1:1000), phospho-STAT1 (Cell Signaling Technology, 1:1000), STAT6 (Cell Signaling Technology, 1:1000), phospho-STAT6 (Cell Signaling Technology, 1:1000), JAK1 (Cell Signaling Technology, 1:1000), phospho-JAK1 (Cell Signaling Technology, 1:1000), Annexin A1 (Abcam, 1:2000), ERK1/2 (Cell Signaling Technology, 1:1000), phospho-ERK1/2 (Cell Signaling Technology, 1:1000), p38 (Cell Signaling Technology, 1:1000), phospho-p38 (Cell Signaling Technology, 1:1000), c-Jun (Cell Signaling Technology, 1:1000), phospho-c-Jun (Cell Signaling Technology, 1:1000), p65 (Cell Signaling Technology, 1:1000), phospho-p65 (Cell Signaling Technology, 1:1000), PI3K (Cell Signaling Technology, 1:1000), phospho-PI3K (Cell Signaling Technology, 1:1000), AKT (Cell Signaling Technology, 1:1000), phospho-AKT (Cell Signaling Technology, 1:1000), p53 (Abcam, 1:400), Bcl-2 (Abcam, 1:1000), Bax (Cell Signaling Technology, 1:1000), Fas (Cell Signaling Technology, 1:1000), cleaved-caspase 3 (Cell Signaling Technology, 1:1000), TBP (Proteintech Group, 1:1000), and β-actin (Cell Signaling Technology, 1:5000).

Flow cytometry analysis

Flow cytometry analysis was performed to assess the intracellular expression of IL-2, IFN-γ, TNF-α, IL-4, and IL-10, nuclear expression of T-bet, GATA3, and Ki67,

and surface expression of ST2 and annexin A1 by T cells treated with indicated conditions, as well as the intracellular expression of MPO and surface expression of ST2, MHC-I and MHC-II by CRC cells treated with indicated conditions. For Xenograft-derived T cell staining, tumor tissues were minced, and the tissue homogenate was then filtered through a 70-μm filter to isolate single cells. T cells were identified with CD3, CD4, and CD8. To examine the expression of IL-2, IFN-γ, TNF-α, IL-4, IL-10, T-bet, GATA3, and Ki67 by T cells, isolated T cells were stained with anti-CD3, anti-CD4, anti-CD8, anti-7AAD, anti-IL-2, anti-IFN-γ, anti-TNF-α, anti-IL-4, anti-IL-10, anti-T-bet, anti-GATA3, and anti-Ki67. For cytokine detection, cells were stimulated with PMA and ionomycin (Sigma-Aldrich, Atlanta, GA, USA) in the presence of a protein transport inhibitor brefeldin A (BioLegend, San Diego, CA) for 6 h, and then stained with fluorochrome-conjugated primary antibodies. All the antibodies were purchased from BD Bioscience, San Diego, USA. Intracellular staining and surface staining were performed as previously described [1]. Nuclear staining was performed by using a Transcription Factor Staining Buffer Set (Thermo Scientific, Waltham, MA, USA).

Apoptosis assay

CRC cells treated in indicated conditions cells were harvested and washed with ice-cold PBS twice. And then the cells were suspended in the Annexin V-binding buffer to a final concentration of 10^6 cells/ml and stained with ant-human Annexin V antibody (BD) and propidium iodide (BD) at 4 °C. The apoptosis rate was analyzed by flow cytometry in 15min.

CCK8 assay

3×10^3 CRC cells per well in 100ul were added to the 96-well plate and cultured at 37 °C. Cell viability was assessed by CCK8 kit (Dojindu, Tokyo) at indicated time points. A batch of cells was stained with 10 μL of CCK8 regent at 37 for 1 h and the absorbance at 450 nm wavelength was measured to calculate the number of viable cells.

ELISA

Secretion of the IL-33, CXCL10, and CXCL13 in the serum or culture supernatants was examined using commercially available ELISA kits (BioLegend, San Diego, CA and Abcam, Cambridge, MA, USA) according to the manufacturer's protocol.

GSH detection

The supernatants of CRC cells treated by indicated conditions were collected. The level of GSH in the supernatants was examined using a GSH detection kit (Jiangsu, KeyGEN Biotech, China) according to the manufacturer's protocol.

Transient transfection

Transfections were performed using the Lipofectamine 3000 Kit (Invitrogen Carlsbad, CA, USA) according to the manufacturer's instructions. Cells were grown to 50% to 60% confluence in 6-well plates and transfected with plasmid containing siRNAs. Cells were harvested 48 h after transfection for subsequent confirming the transfection efficiency by western blot.

Lentivirus vector construction and cell infection

All recombinant lentiviral vectors and control vectors were constructed by GenePharma (Shanghai, China). Lentiviral infection was performed by adding virus solution to the cells in the presence of 5 μg/mL polypropylene (Sigma-Aldrich). After infection for 72 h, cells were selected in the presence of 2 μg/mL puromycin, and cells resistant to puromycin were collected and cultured. The construction of stable cell lines was completed.

T cell migration assay

T cell migration was assessed by using a 24-well Transwell System with 3.0 μm polycarbonate membranes (Corning, NY, USA). A total of 2.0×10^6 T cells in 100 μl of serum-free RPMI 1640 medium and added to the upper chamber. 600 μl medium alone and with rhCXCL10 (10 ng/mL, Peprotech), rhCXCL13 (10 ng/mL, Peprotech) or with 50% indicated culture supernatants from CRC cells was added to the lower

chamber. After incubation at 37 °C for 2 h, the cells that migrated into the lower chamber were harvested and counted using a hemocytometer. The negative control was T cells that migrated toward RPMI 1640 alone. The chemotactic index was calculated as the ratio of the number of T cells that migrated to conditioned medium divided by the number of those migrating to RPMI 1640 alone.

Statistical analysis

Statistical analyses were performed using SPSS version 19.0 or Prism 8 (Graph Pad Software Inc.). Data are expressed as the mean ± SD according to the distribution level. Differences between groups with normally distributed continuous variables were analyzed using independent-sample or paired t-test. The association between the expression levels of two markers was analyzed using Pearson's correlation coefficient. The Kaplan-Meier method and log-rank test were used to plot survival curves and analyze differences in survival time between patient subgroups. Cox's proportional hazards regression model was used to evaluate the prognostic value of the risk factors. In all analyses, a two-tailed $P < 0.05$ was considered statistically significant, *$P < 0.05$, **$P < 0.01$, ***$P < 0.001$, ****$P < 0.0001$.

References

1. Chen CL, Wang Y, Huang CY, Zhou ZQ, Zhao JJ, Zhang XF, et al. IL-17 induces antitumor immunity by promoting beneficial neutrophil recruitment and activation in esophageal squamous cell carcinoma. Oncoimmunology. 2017;7(1):e1373234.

2. Song M, He J, Pan QZ, Yang J, Zhao J, Zhang YJ, et al. Cancer-Associated Fibroblast-Mediated Cellular Crosstalk Supports Hepatocellular Carcinoma Progression. Hepatology. 2021;73(5):1717-35.
